# Suppressing Tymovirus replication in plants using a variant of ubiquitin

**DOI:** 10.1371/journal.ppat.1012899

**Published:** 2025-01-27

**Authors:** Anuradha De Silva, Kihun Kim, John Weiland, Jihyun Hwang, Jacky Chung, Higor S. Pereira, Trushar R. Patel, Joan Teyra, Ankoor Patel, Mohammed M. Mira, Mazdak Khajehpour, Melvin Bolton, Claudio Stasolla, Sachdev S. Sidhu, Brian L. Mark

**Affiliations:** 1 Department of Microbiology, Faculty of Science, University of Manitoba, Winnipeg, Manitoba, Canada; 2 Sugarbeet and Potato Research Unit, Edward T. Schafer Agricultural Research Center, USDA Agricultural Research Services, North Dakota, United States of America; 3 School of Pharmacy, University of Waterloo, Ontario, Canada; 4 Alberta RNA Research and Training Institute, University of Lethbridge Department of Chemistry and Biochemistry, University of Lethbridge, Lethbridge, Alberta, Canada; 5 Icosagen Cell Factory OÜ, Tartu, Estonia; 6 Department of Plant Science, Faculty of Agriculture, University of Manitoba, Winnipeg, Manitoba, Canada; 7 Department of Chemistry, Faculty of Science, University of Manitoba, Winnipeg, Manitoba, Canada; China Agricultural University, CHINA

## Abstract

RNA viruses have evolved numerous strategies to overcome host resistance and immunity, including the use of multifunctional proteases that not only cleave viral polyproteins during virus replication but also deubiquitinate cellular proteins to suppress ubiquitin (Ub)-mediated antiviral mechanisms. Here, we report an approach to attenuate the infection of *Arabidopsis thaliana* by Turnip Yellow Mosaic Virus (TYMV) by suppressing the polyprotein cleavage and deubiquitination activities of the TYMV protease (PRO). Performing selections using a library of phage-displayed Ub variants (UbVs) for binding to recombinant PRO yielded several UbVs that bound the viral protease with nanomolar affinities and blocked its function. The strongest binding UbV (UbV3) candidate had a EC_50_ of 0.3 nM and inhibited both polyprotein cleavage and DUB activity of PRO *in vitro*. X-ray crystal structures of UbV3 alone and in complex with PRO reveal that the inhibitor exists as a dimer that binds two copies of PRO. Consistent with our biochemical and structural findings, transgenic expression of UbV3 in the cytosol of *A*. *thaliana* suppressed TYMV replication *in planta*, with the reduction in viral load being correlated to UbV3 expression level. Our results demonstrate the potential of using UbVs to protect plants from tymovirus infection, a family of viruses that contain numerous members of significant agricultural concern, as well as other plant viruses that express functionally related proteases with deubiquitinating activity.

## Author summary

Plant viruses pose significant threats to agricultural crops, and their presence in northern latitudes stands to increase as pests, including those vectoring viruses and pathogens, advance poleward due to continued global warming. Developing effective and environmentally responsible antiviral control measures for plants could be used to counteract the climate-related spread of plant viruses. Antiviral strategies based on genetic engineering have enhanced plant virus resistance, and some have met with commercial success. To further broaden the diversity of transgenic-based antiviral approaches for agricultural use, we have developed a protein-based inhibitor that can be expressed in plants to selectively block viral protease activity and suppress virus replication *in planta*. We demonstrate that ubiquitin variants (UbVs) can be rapidly isolated from a phage display library and used to potently and selectively inhibit the activity of the TYMV protease (PRO) from the model plant virus Turnip Yellow Mosaic Virus (TYMV). Moreover, transgenic expression of a UbV in the model plant *Arabidopsis thaliana* reduced TYMV viral load in challenged plants. Our study provides proof-of-principle that UbVs can be identified and used to impart resistance to Tymovirus infections in plants and potentially other plant viruses that code for proteases with deubiquitinating activity.

## Introduction

It is estimated that food production may need to increase by up to 70% over the next 30 years to sustain the world’s growing population [1]. How to increase crop production on a decreasing amount of arable land is arguably one of the most pressing challenges of our time. Central to this challenge will be the discovery of ecologically sustainable ways to protect crops from disease. Current losses of major crops from biotic stresses such as fungi, viruses and bacteria are estimated at 20–40% [2], which stands to increase in northern latitudes as pests and pathogens advance poleward due to global warming [3]. Among these pathogens, viruses inflict significant biotic stress on plants, with crop-related loss estimates approaching thirty billion U.S. dollars in loss annually [4]. Losses from viral infection are second only to those caused by fungi [5] and currently account for ~47% of emerging infectious diseases in plants [6].

There are many steps in the replication cycle of viruses that can be targeted for inhibitor development, several of which have been exploited to create effective antiviral drugs for human use [7]. Of these, viral proteases have been particularly successful targets since they are essential for viral replication and amenable to drug development. The genomes of animal and plant RNA viruses often contain long open reading frames from which large polyproteins are expressed and subsequently cleaved into multiple functional units by one or more viral proteases [8]. These proteases are found as domains within the polyproteins and cleave the polyprotein apart either in *cis* or *trans* [8]. These cleavage events produce activated proteins supporting virus replication, movement between cells and within the plant, encapsidation of the viral genome, and transmission of mature viruses by vectors. Since polyprotein processing is essential to virus replication, proteases involved in their cleavage are excellent antiviral drug targets [7].

Interestingly, several virus families from plants and animals code for proteases that not only catalyze polyprotein cleavage but also act as deubiquitinases (DUB) to usurp ubiquitin (Ub)-dependent host antiviral responses [9–15]. Viral deubiquitinases (vDUB) recognize the C terminus of Ub (^73^LRGG^76^) and cleave the isopeptide bond downstream of the terminal Gly76 to remove Ub from cellular proteins [16,17]. Ub is often added to cellular proteins as poly-Ub chains, which are assembled by linking the C terminus of Ub to one of 7 internal lysines of the next Ub in the chain [18]. Each linkage type provides a unique signal to the cell, for example, K48- and K63-linked Ub chains, being used to target proteins for proteasomal degradation and activate host antiviral pathways, respectively [19]. Given the roles of viral DUBs in polyprotein processing and suppression of Ub-mediated antiviral responses [11,20], blocking the activity of these enzymes presents a promising antiviral strategy. To explore this possibility, we have employed an approach that identifies highly selective and potent protein-based inhibitors of vDUBs from phage-displayed libraries of ubiquitin variants (UbVs) [21]. Previously, we demonstrated that UbVs can potently inhibit vDUBs from several viruses, including the Middle East Respiratory Syndrome coronavirus (MERS-CoV) [22], Crimean Congo Hemorrhagic Fever Virus (CCHFV) [22] and Severe Acute Respiratory Syndrome Coronavirus 2 (SARS-CoV-2) [23]. Blocking vDUB activity with UbVs interferes with their polyprotein processing and deubiquitinating activity, which in turn interferes with virus replication [22,23].

Though roughly half (~45%) of all plant-infecting viruses encode proteases [8], relatively little has been done to exploit them as targets for infection control compared to animal viruses. Since UbVs are proteins, we wanted to know if their expression in plants could be used to protect plants from pathogenic viruses that rely on a vDUB for polyprotein processing and cellular immune evasion. We explored this idea using the model virus Turnip Yellow Mosaic Virus (TYMV), which encodes a vDUB known as PRO that suppresses Ub-dependent antiviral responses and is vital to virus replication as a protease that processes the viral polyprotein [16,24–27]. TYMV is a single-stranded RNA plant virus that can cause significant damage to plants, particularly turnips and other members of the *Brassicaceae* family [28–30]. Numerous other vDUBs have been identified across diverse and distantly related plant and plant-associated virus lineages, such as Maize rayado fino virus (*Marafivirus*) [9], Beet necrotic yellow vein virus (*Benyviruses*) [10], and Rice stripe tenuivirus (*Tenuivirus*) [31], and the recently characterized Tymo-like viruses in mosquitoes and insects known to obtain sugar from floral nectar [32].

We used a library containing billions of phage-displayed UbVs to select several distinct UbVs that bound tightly to recombinant PRO. The tightest binding UbV (UbV3) was chosen for further study and was found to block both polyprotein cleavage and DUB activity of PRO *in vitro*. X-ray structures of UbV3 in isolation and bound to PRO reveal that the inhibitor exists as a dimer that binds two copies of PRO. Consistent with our biochemical and structural findings, transgenic expression of UbV3 in the cytosol of *A*. *thaliana* suppressed TYMV replication *in planta*, with the reduction in viral load being correlated to UbV3 expression levels. Our results demonstrate the potential of using UbVs to protect plants from tymovirus infection, a family of viruses that contains numerous members of significant agricultural concern.

## Results

### Screening of ubiquitin variants that bind with TYMV

**Fig 1:**
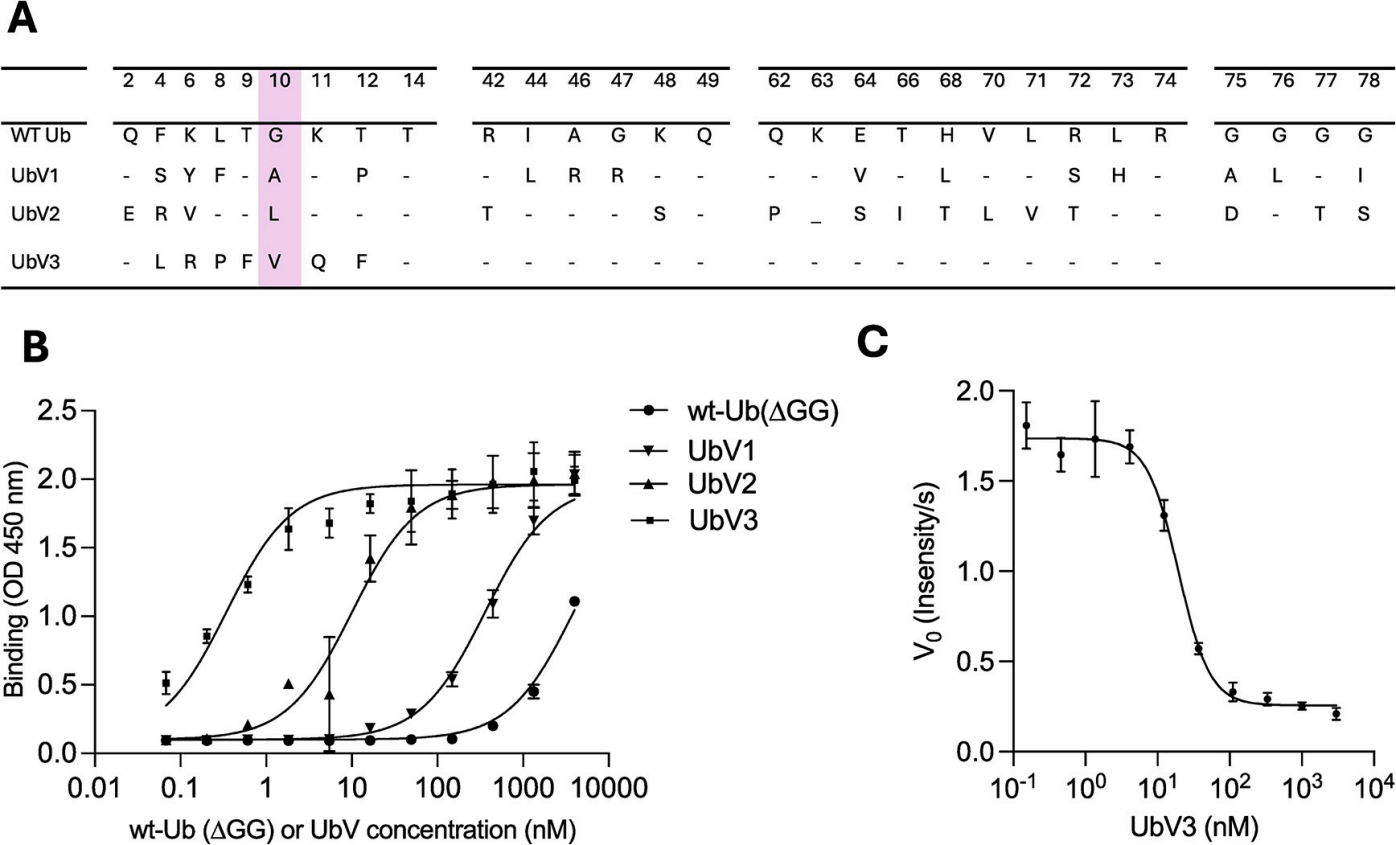
Sequences and binding of UbVs to PRO. (A) Sequence alignment of WT Ub with UbVs 1, 2 and 3. Dashes indicate identity with the WT. The highlighted (magenta) substitutions at position 10 are known to cause dimerization of UbVs [33], which was confirmed for UbV3 by X-ray crystallography (Fig 5). (B) The following half-maximal binding concentrations (EC_50_) of UbVs to PRO were determined as described [68]: >1000 nM (WT Ub, circles), 560 nM (UbV1, inverted triangles), 11 nM (UbV2, triangles), 0.3 nM (UbV3, squares). (C) Inhibition of PRO by UbV3 shown as a dose-response curve using Ub-AMC as a substrate. The IC_50_ value (19.5 ± 2.7 nM) was determined as the concentration of UbV that reduced deubiquitination activity by 50%.

PRO was expressed as a soluble and active DUB in *Escherichia coli* with its N-terminus fused to a removable (HRV-3C cleavage site) His_6_ affinity tag or GST affinity tag (S1 Fig). The GST-PRO fusion protein was used in binding selections with a phage-displayed library of UbVs, as described previously for other DUB proteins [21–23], and His_6_-PRO was used for structural studies. Three UbVs were identified that exhibited selective and potent binding to PRO. Fig 1 shows the resulting sequences and binding affinities of UbVs to the PRO protease. Based on binding data using purified recombinant UbVs 1, 2 and 3 (Fig 1B), UbV3 was found to have the tightest binding with PRO (EC_50_ = 0.3 nM). The dose-response curve revealed that UbV3 effectively inhibited the hydrolysis of Ub-AMC, with an IC_50_ value of 19.5 nM, further confirming its potent inhibitory effect on the protease (Fig 1C). Notably, all three UbVs contained substitutions at position 10, which is known to cause UbV dimerization [33] and was later confirmed by X-ray crystallography as described below. Given its binding potency, UbV3 was selected for further studies. Recombinant UbV3 with N-terminal His_6_ and FLAG tags was expressed in *E*. *coli*. The His_6_ tag was used for purification by affinity chromatography with Ni-NTA resin, and the FLAG tag was used for detection by western blotting, as described below.

**Fig 2:**
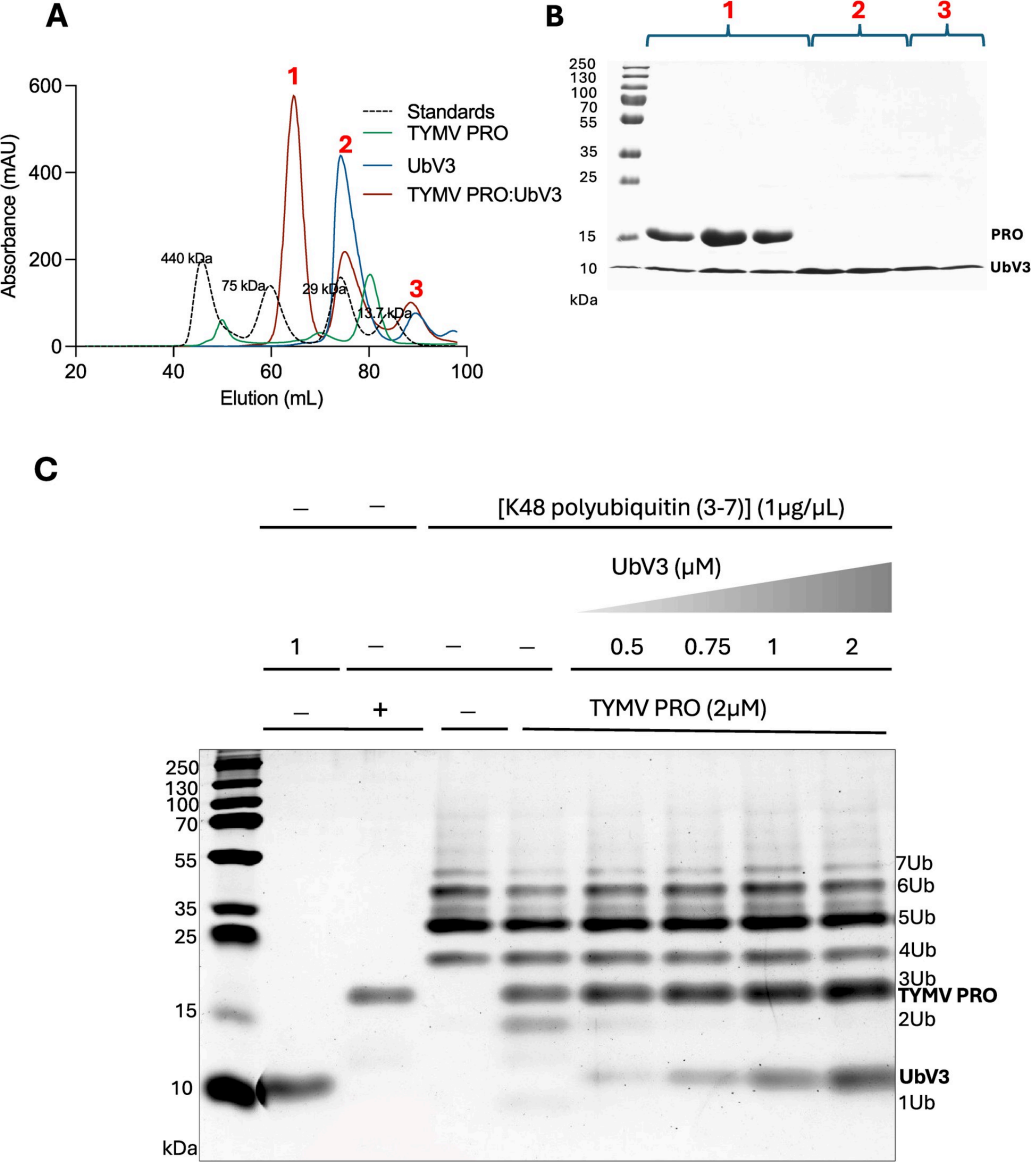
UbV3 interaction with TYMV PRO and inhibition of K48-linked polyUb chain cleavage by TYMV PRO. (A) Gel filtration of TYMV PRO bound to UbV3 is shown in red (TYMV PRO and UbV3 were mixed at a 1:2 ratio). The elution profile of UbV3 in the absence of TYMV PRO is shown in blue. UbV3 appears to exist primarily as a homodimer (peak 2) in equilibrium with its monomeric form (peak 3). The elution profile of TYMV PRO without UbV3 is shown in green. Molecular mass standards are shown in the black dashed line. (B) 12% SDS-PAGE of the peaks collected by gel filtration in panel A. Lanes 1, 2 and 3 are TYMV PRO bound to UbV3 (peak 1 at ~65 ml, shown in red in panel A). Lanes 4, 5 and 6, 7 are proteins from peaks 2 and 3 in red at ~75 ml and ~88 ml, respectively, and are dimeric and monomeric UbV3, respectively. (C) Purified TYMV PRO (2 μM) was incubated for 30 minutes at 25°C with 1 μg of K48-linked polyUb chains in the presence of increasing concentrations of UbV3 in a final volume of 12 μL. The DUB activity was inhibited by UbV3, with DUB activity significantly reduced with a 2:1 molar ratio of TYMV PRO and UbV3. PolyUb chains were visualized using TRIS-Tricine SDS-PAGE (10%) and detected using a Pierce Silver Stain Kit.

To further explore the interaction between PRO and UbV3, as well as the structural complex they form, PRO was mixed with an excess of UbV3 (1:2 molar ratio) and the complex purified by gel filtration chromatography (Fig 2A and 2B). Gel filtration results suggested that UbV3 might be forming a homodimer that bound two copies of PRO, which was confirmed by X-ray crystallography and small-angle X-ray scattering (see below).

### UbV3 inhibits the DUB and polyprotein cleavage activities of PRO

Previous studies demonstrated that proteins coded by TYMV, including the viral RNA-dependent RNA polymerase, are prone to K48 polyubiquitination by the host cell and subsequent destruction by the Ub proteasome system (UPS) both *in vitro* and *in vivo* [14,25,34]. This cellular defense mechanism is suppressed by the K48-polyUb DUB activity of PRO [15], presumably to enhance replication of the virus, which PRO also assists in the processing of the viral polyprotein [27,35].

To further examine the ability of the UbV3 dimer to block the DUB activity of PRO, we measured its ability to inhibit K48-linked polyUb chain cleavage by PRO (Fig 2C). Indeed, this DUB activity was inhibited by UbV3, with DUB activity significantly reduced with a 2:1 molar ratio of PRO and UbV3 (Fig 2C).

**Fig 3:**
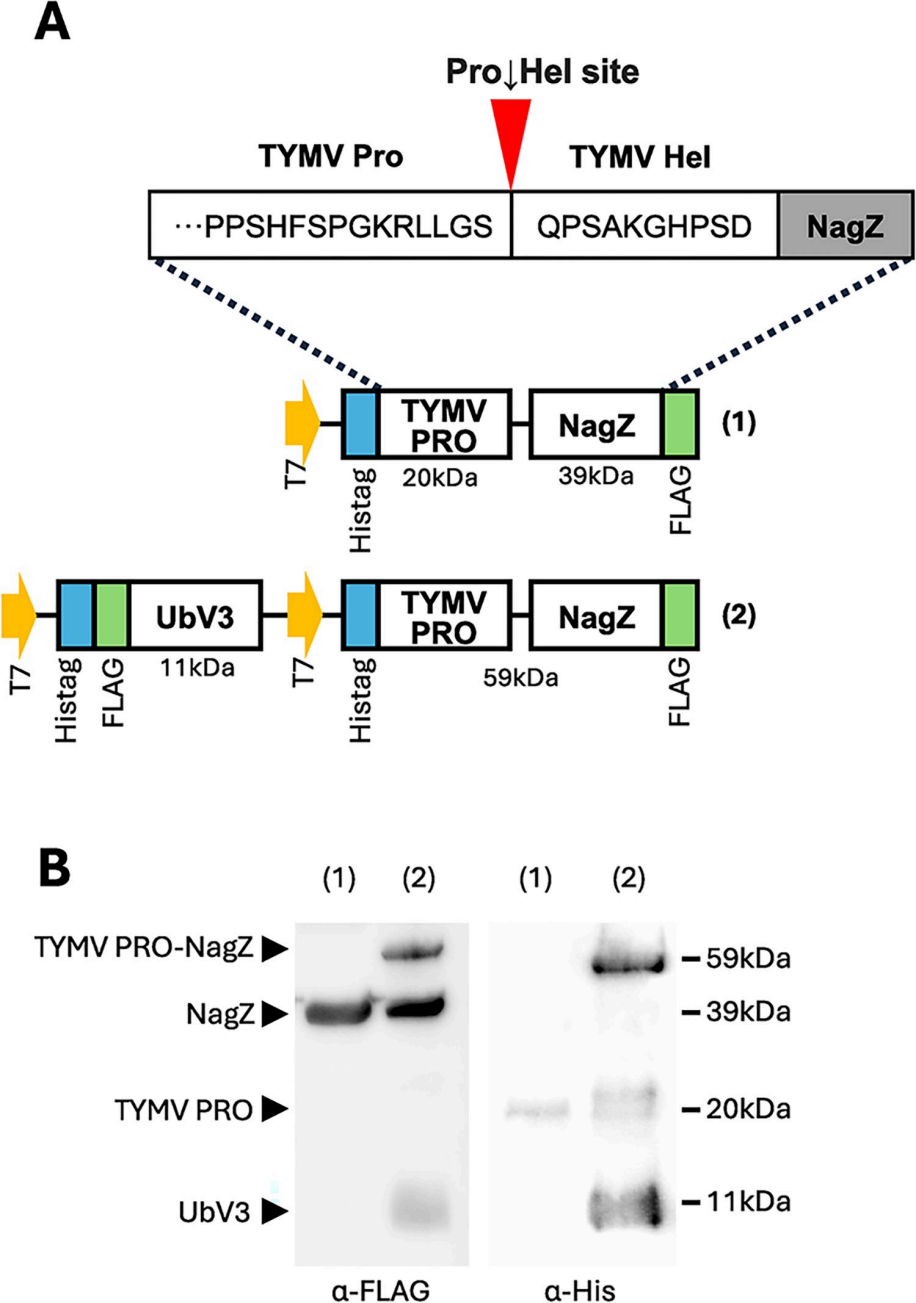
Inhibition of TYMV PRO cleavage by UbV3. (A) Construct design for demonstrating cleavage of the Pro↓Hel site by PRO (A-1) and its inhibition by UbV3 (A-2). (B) Western blot analysis of cell lysates from *E*. *coli* BL21(DE3) expressing PRO-NagZ(A-1) alone, or co-expressing PRO-NagZ with UbV3 (A-2). The gels are representative of 3 independent replicates. Ponceau stains of the blots are shown in S3 Fig copies to demonstrate protein loading consistency for each experiment.

The TYMV viral polyprotein is cleaved at two sites by PRO to release the viral helicase and polymerase [27,36]. We wanted to determine if UbV3 blocks this polyprotein cleavage activity *in vitro* (Figs 3 and S3). To achieve this, we created an artificial substrate that included the cleavage site in the TYMV polyprotein between the PRO domain and helicase (Pro↓Hel site). The Pro↓Hel site was inserted between a copy of PRO and the glycosidase NagZ [37] and was expressed in the presence or absence of UbV3 (Fig 3A). The PRO-NagZ fusion protein served as a reasonable substrate to assess cleavage of the Pro↓Hel site by PRO and the inhibition of this activity by UbV3, since its cleavage by PRO could be readily monitored by the appearance of intact PRO-NagZ (~59 kDa), or free PRO (~20 kDa) and NagZ (~39 kDa) by Western blot (Fig 3B).

We confirmed the Pro↓Hel site within the PRO-NagZ fusion protein is cleaved by PRO to release free PRO and NagZ proteins (Fig 3B). Whether this occurs in *cis* or *trans* was not determined. Notably, co-expression of the PRO-NagZ fusion with UbV3 inhibited cleavage of the Pro↓Hel site as noted by the presence of intact PRO-NagZ protein (Fig 3B), suggesting that UbV3 also impairs PRO-mediated processing of the viral polyprotein during virus replication.

### Evaluating off-target inhibition of cellular plant DUBs by UbVs

**Fig 4:**
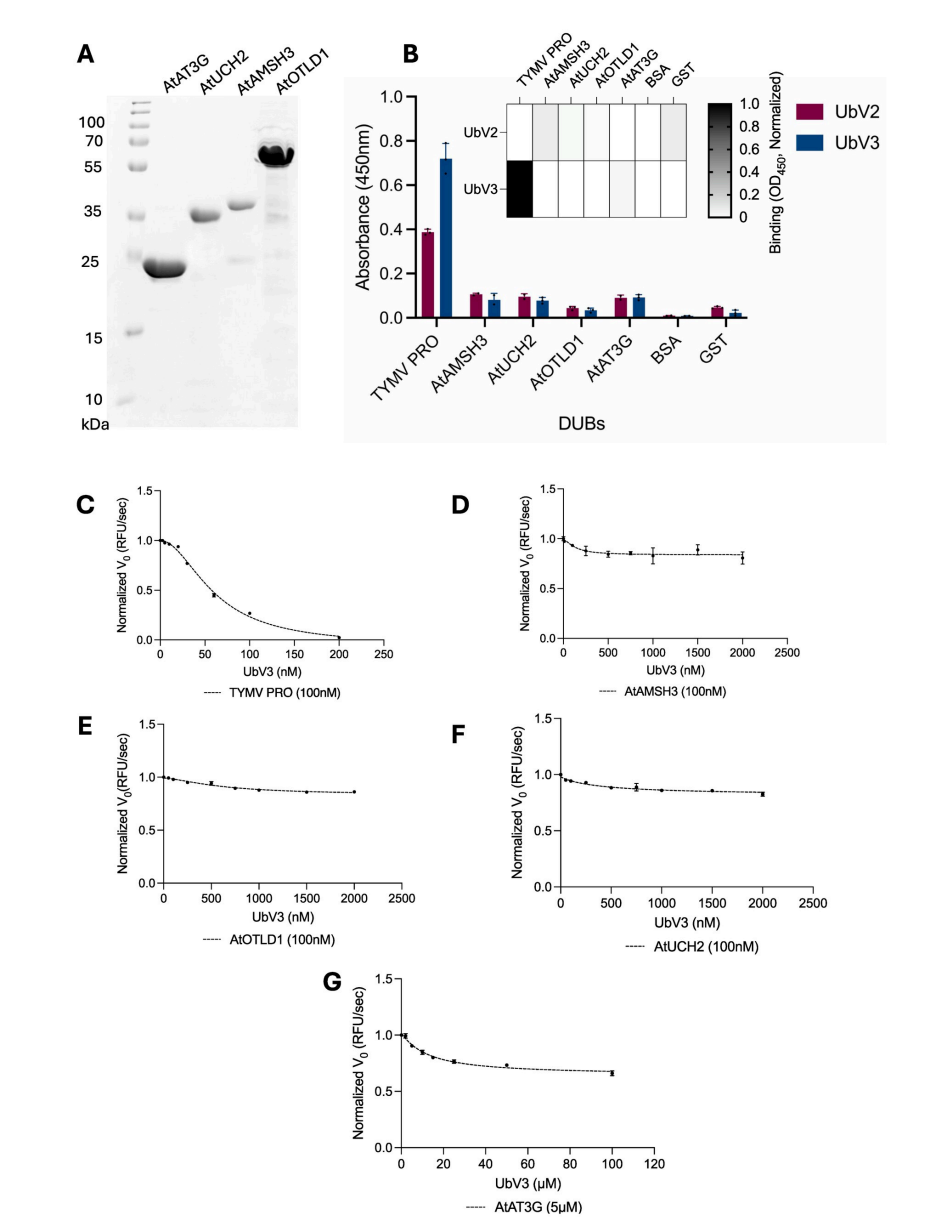
The binding potency of UbVs against TYMV PRO and *A*. *thaliana* DUBs. (A) SDS-PAGE gel demonstrating the purity of soluble recombinant DUB domains of 4 different families of *Arabidopsis* plant DUBs: AtAT3G (26 kDa), AtUCH2 (40 kDa), AtAMSH3 (47 kDa) and AtOTLD1 (57 kDa). (B) Binding specificities of the most potent UbVs (UbV2 and UbV3) that block TYMV PRO activity. The histogram shows the absorbance values of UbV3 binding with DUBs and negative control proteins, and the heat map shows the normalized absorbance values of the interactions. The mean value of absorbance at 450 nm is shaded in a grayscale. (C) Inhibition curve of TYMV PRO (100nM) with 0-200nM concentrations of UbV3. (D-G); inhibition curves of AtAMSH3 (100nM) (D), AtOTLD1 (100nM) (E), AtUCH2(100nM) (F), with 0- 1000nM concentrations of UbV3. (G) Inhibition curve of AtAT3G with 0–50μM concentrations of UbV3. The predicted AtAT3G enzyme is only a moderately active against Ub-AMC; therefore, 5μM of enzyme concentration and 0–100μM concentrations of UbV3 were used to analyze the inhibitory effect of UbV3 against AtAT3G. The data display the initial rate ± SED of 3 replicates, and data were collected at 25°C for 3 hours. The IC_50_ values were calculated as follows; TYMV PRO (IC_50_ = 60±0.06nM), [AtUCH2, AtAMSH3 and AtOTLD1 (IC_50_ >2000nM) and predicted DUB AtAT3G (IC_50_ >100μM). Progress curves were fitted to a non-linear regression equation (y = y_0_+ae^bx^) to determine the initial rate of each reaction using SigmaPlot software (initial rate = a × b, refer to **S3 Table**).

To determine if UbVs selected by phage display exhibited any non-specific interaction with cellular DUBs, the UbVs were assessed against a panel of recombinant plant DUBs cloned from the model plant species *Arabidopsis thaliana*. Synthetic open reading frames of *A*. *thaliana* DUBs were ligated into pGEX-6P-1, pET19b or pET28a vectors for expression and subsequent purification from *E*. *coli*. The recombinant *A*. *thaliana* DUBs were purified to homogeneity (Fig 4A) to generate a panel that represented 4 of the 5 major DUB families found in plants, as follows: AtAMSH3 (JAMM family) [38], AtAT3G (predicted plant deubiquitinase / MJD family) [39], AtUCH2 (UCH family) [40], and AtOTLD1 (OTU family) [41]. While attempts were made to include representatives from the UBP family, we were unable to express members from this family as soluble proteins in *E*. *coli*. Of the families for which representative plant DUBs were produced, each was confirmed to be active using Ub-AMC as substrate (S2 Fig). Notably, the MJD family DUB AtAT3G (NCBI: At3G54130) was only predicted to be a DUB based on sequence alignments [39], but nonetheless, we verified it to be a functional DUB and included it in our *A*. *thaliana* DUB panel.

The ability of the UbVs to bind to members of the *A*. *thaliana* panel of DUBs, or to TYMV PRO and negative controls GST and BSA was measured by ELISA. Consistent with the high selectivity we previously observed for UbVs selected against animal vDUBs [22], we found that UbVs selected against PRO did not bind appreciably to any of the DUBs from *A*. *thaliana*, or negative control proteins (Fig 4B). In addition to ELISA-based binding assays, we also enzymatically probed the selectivity of UbV3 for PRO versus the panel of *A*. *thaliana* DUBs. Using Ub-AMC as substrate, the activity of each DUB was measured in the presence of increasing amounts of UbV3. Kinetic profiles were then fitted to a non-linear regression equation to obtain initial rates for each reaction (Fig 4 and S3 Table). The initial rates were plotted against UbV3 concentrations to determine IC_50_ values. Consistent with the ELISA protein binding assays, activity-based assays demonstrated that even at very high concentrations (>40-fold greater than needed to reduce PRO activity by half), UbV3 had minimal effect on the activity of the *A*. *thaliana* DUBs, yet it potently blocked PRO activity (Fig 4**)**. Together, the above binding and inhibition assays confirmed that UbV3 is a highly specific and potent inhibitor of PRO and suggested that its transgenic expression in *A*. *thaliana* could potentially suppress TYMV replication without perturbing the cellular Ub system, and thus not adversely affect plant physiology.

### G10V substitution is essential for the dimerization of UbV3

**Fig 5:**
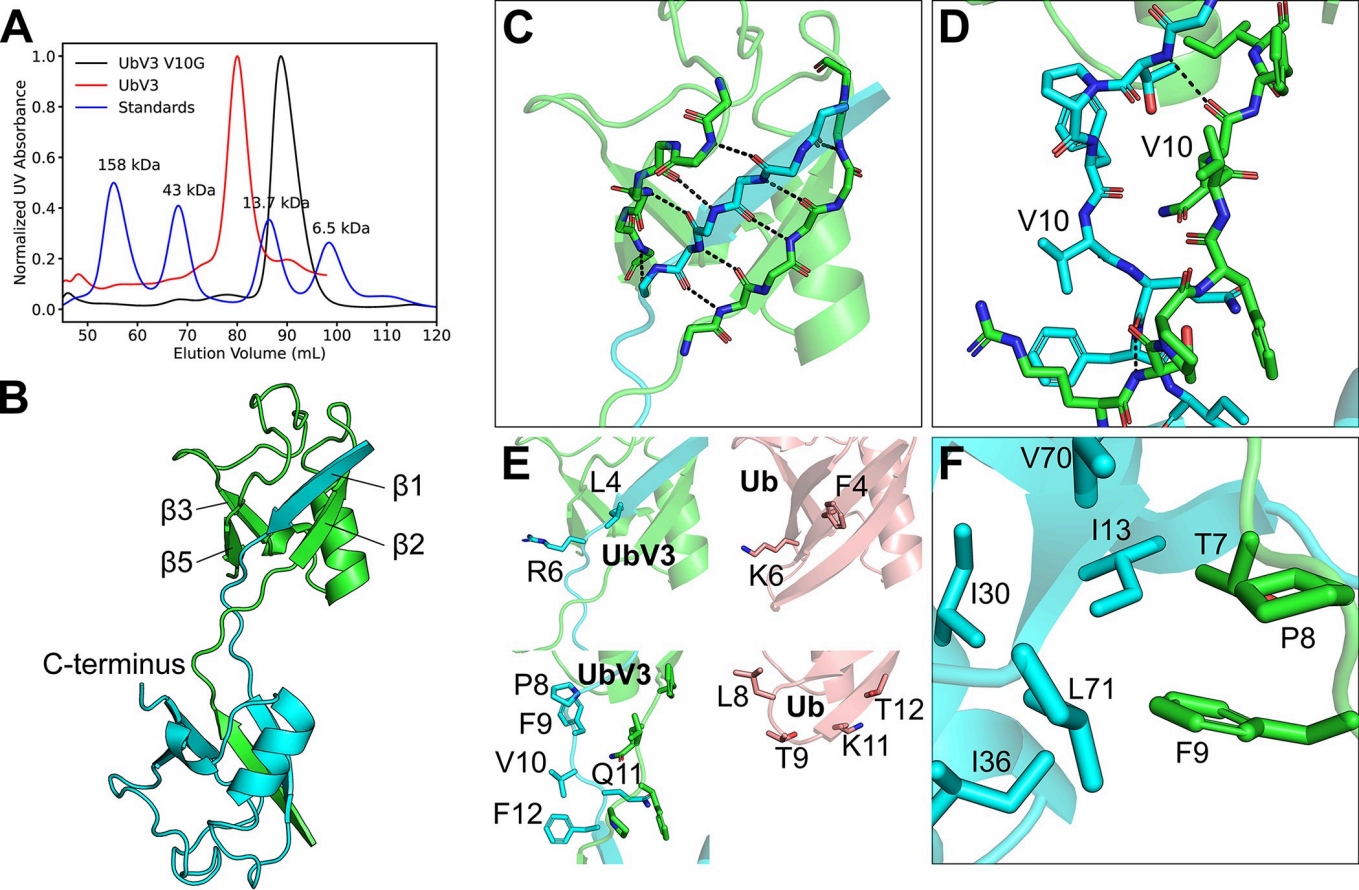
Crystal structure of the UbV3 dimer. (A) Gel filtration chromatography profile of UbV3 V10G construct and UbV3. With the aid of standards, UbV3 V10G was clearly identified as monomer instead of being a dimer like UbV3. (B) Cartoon representation of the UbV3 dimer. (C) Backbone hydrogen bond network near strand β1 that holds the UbV monomer together through strand exchange. (D) β1-β2 loop structure. (E) Substitutions in UbV3 compared to wild type Ub. UbV3 chains are colored as cyan and green, and ubiquitin (PDB ID: 1UBQ) is colored as salmon. Substitutions around strand β1 are described on top panel, and substitutions around β1-β2 loop are described on bottom panel. (F) Hydrophobic sidechains near I36 patch interact with β1-β2 loop region.

To gain insight into how UbV3 blocks PRO activity, we determined the X-ray crystal structures of UbV3 alone and bound to PRO (S1 Table). Ubiquitin variants with substitutions at position 10 have been reported to dimerize [33,42]. Substitution at position 10 (glycine in wildtype Ub) to valine or alanine causes the N-terminal b-sheet of ubiquitin to unfold and participate in dimerization interactions [33]. G10 is located in a loop between strands β1 and β2 of Ub, and its substitution to valine or alanine destabilizes the β1-β2 loop, causing strand β1 to engage with strands β2 and β5 of another UbV3 monomer to form a stable dimer with β1 strands exchanged [33]. Reverting V10 back to a glycine in UbV3-(V10G) appeared to abolish dimerization to yield a monomeric protein (Fig 5A), suggesting that G10V is a substitution that favours UbV3 dimerization.

An X-ray structure of UbV3 revealed that it indeed forms a dimeric structure, as suggested from the chromatography studies (Fig 5B). Strand β1 of each UbV3 monomer was exchanged to form hydrogen bonds with strand β2 and strand β5 of the partnering UbV3 monomer within the dimer. The resulting β-sheet formed by these strands holds the dimer together (Fig 5C). The extended loops between strands β1 and β2 that link the monomers together do not show any appreciable interactions with each other, suggesting structural flexibility in this region (Fig 5D). There are 7 amino acid substitutions in UbV3 with respect to WT Ub (F4L, K6R, L8P, T9F, G10V, K11Q, T12F) (Fig 1A). In WT Ub, the sidechains of F4 and K6 project outward from the protein, and the conservative substitutions at these positions in UbV3 (F4L, K6R) do not affect the fold and would only minimally affect hydrophobicity or polarity in this region (Fig 5E top). Just downstream of strand β1, L8 substituted to proline in UbV3, where the proline has a cis-peptide bond that enables the backbone atoms to adopt similar positions to that found for the T7-L8 peptide bond of Ub. However, the backbone dihedral angles of F9 of UbV3 were changed dramatically (φ = -83.1°, ψ = 141.3°), compared to that of T9 (φ = -101.4°, ψ = 14.9°) in WT Ub, and those of V10 in UbV3 were also changed (φ = -92.3°, ψ = 129.3°) compared to G10 (φ = 77.4°, φ = 16.5°) of WT Ub (S4 Fig). These angular changes reflect the change of the β1-β2 loop from a type I β-turn in WT Ub to an extension that reaches out to engage with the partnering monomer in the UbV3 dimer (Fig 5B). Substitutions K11Q and T12F did not affect backbone geometry, nor did their sidechains interact with other atoms of the UbV3 dimer (Fig 5E, bottom).

Finally, the sidechain of F9 of each monomer of UbV3 packs closely with several hydrophobic sidechains of other nearby residues (I13, I30, I36, V70, L71) (Fig 5F). Known as the I36 patch in WT Ub, this patch participates in protein-protein interactions [43], including binding of Ub to PRO [26]. The site is conserved in UbV3, and prior to determining the X-ray crystal structure of UbV3 bound to PRO, we speculated that its participation in binding the protease may be enhanced by the T9F substitution. Indeed, the pyrrolidine ring of P8 and aromatic ring of F9 are ~3 Å apart and appear to form a C-H/π interaction that places the F9 sidechain in an orientation that helps stabilize the extended conformation of the β1-β2 loop along with the G10V substitution to form the UbV3 dimer.

### UbV3 dimerization drives PRO inhibition

PRO binds UbV3 in solution as a single complex that can be readily purified by gel filtration chromatography (Fig 2A and 2B), and its mass (~50 kDa) suggested that two copies of PRO were bound to one UbV3 dimer (S5 Fig). This was not surprising considering that the UbV3 dimer has two solvent-exposed C-terminal tails (right after strand β5) to which independent copies of PRO could bind (Fig 5B). After several failed attempts to crystallize the complex, we ultimately succeeded by covalently linking UbV3 to PRO by modifying the C-terminal glycines of UbV3 with 3-bromopropylamine as we have done previously to generate stable complexes of vDUBs bound to Ub [44,45].

**Fig 6:**
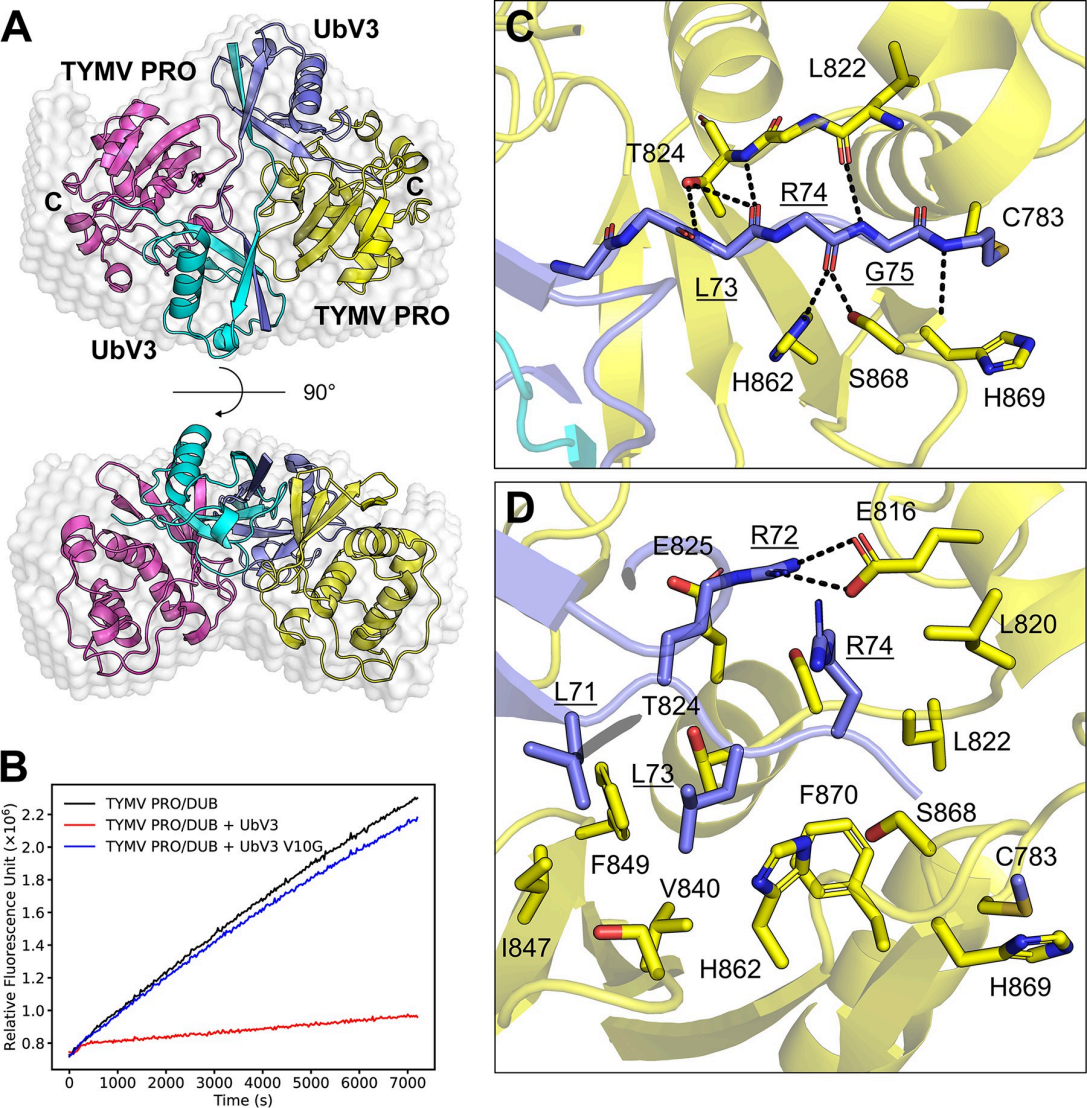
Crystal structure of TYMV PRO in complex with the UbV3 dimer. (A) 2:2 complex of TYMV PRO and UbV3 molecules are present in 1 asymmetric unit. TYMV PRO chains are colored magenta and yellow, UbV3 chains are colored cyan and blue. N and C termini of UbV3 chains are labelled. *ab initio* SAXS envelope of the non-covalent bonded complex is represented as transparent white spheres and shows that the X-ray structure and solution structure are consistent. Covalent linkages are labeled as C, as the C-terminus of UbV3 was covalently linked to TYMV PRO active site C783. (B) UbV3 V10G has a dramatically reduced ability to inhibit TYMV PRO/DUB activity compared to the same amount of UbV3, as identified by a Ub-AMC cleavage assay. (C) Backbone atoms of the C-terminal tail of UbV3 (^71^LRLRG^75^) lie in the active site to form hydrogen bonds with TYMV PRO. (D) Polar interactions between UbV3 and TYMV PRO. Possible interactions are indicated by a dashed line with distance. Residues of UbV3 are labeled with underline, and the key residues of TYMV PRO active site are labelled.

As verified below, the modification yielded covalent linkages between the C-termini of the monomers within UbV3 to the active site cysteines (C783) of bound copies of PRO, which enabled crystallization of the complex for structural analysis (Fig 6A). An *ab initio* envelope determined by small angle X-ray scattering (SAXS) of the non-covalently bonded complex is consistent with the X-ray structure of the covalently bound complex, suggesting the X-ray structure is representative of the non-covalent complex as it exists in solution (Fig 6A).

The X-ray crystal structure of the PRO-UbV complex clearly revealed two copies of PRO bound to one UbV3 dimer (Fig 6A). Each monomer of the UbV3 dimer binds a copy of PRO with a binding mode like that of WT Ub when comparing the complex to the previously determined structure of TYMV PRO bound to Ub [26] (PDB ID: 6YPT) (RMSD of Cα = 0.48 Å for UbV3 versus Ub, RMSD of Cα = 0.97 Å for PRO bound to UbV3 versus Ub) (S6 Fig). Interestingly, dimerization of UbV3 increases the size of a productive binding interface with PRO that does not involve the PRO active site. Reverting V10 back to glycine caused UbV3-(V10G) to prefer a monomeric state (Fig 5A), and it significantly reduced the ability of UbV3-(V10G) to inhibit PRO activity (Fig 6B), demonstrating that V10 itself and/or UbV3 dimerization is necessary for potent inhibition of PRO activity by UbV3.

The C-terminal tail of each UbV3 monomer lies within the active site cleft of PRO, where the C-terminal propylamine is covalently linked to the sidechain of C783 of PRO as expected, and the interactions between the C-terminal tail of UbV3 (^71^LRLRG^75^) and PRO are consistent with how Ub binds the protease (Fig 6C). Most polar interactions between the C-terminal tail of UbV3 and PRO occur with backbone atoms of UbV3. The amide N and carbonyl O atoms of L73 of UbV3 form hydrogen bonds with Oγ atom (3.1 Å) and amide N atom of T824 (2.7 Å) of PRO, respectively. The carbonyl O atom of R74 is close to the Oγ side chain atom of S868 (2.3 Å) and Nη2 side chain atom of H862 (2.9 Å). The sidechains of UbV3 R72 and PRO E816 also engage in hydrogen bonding interactions. Furthermore, the sidechains of L71 and L73 of UbV3 pack against a hydrophobic surface of PRO comprised of V840, I847, F849 and F870 (Fig 6D).

Beyond the C-termini of the UbV3 monomers, the inhibitor makes additional polar contacts at 3 more sites on each copy of PRO (S7 Fig). The sidechain of UbV3 R6 forms a salt-bridge with the sidechain of PRO E759, whose active site binds to another UbV3 chain. The backbone amide N atom of V10 of UbV3 forms a 2.9 Å hydrogen bond with the carbonyl O atom of PRO P846, and the sidechains of H68 of UbV3 and N760 of PRO also engage in a 2.9 Å hydrogen bonding interaction.

**Fig 7:**
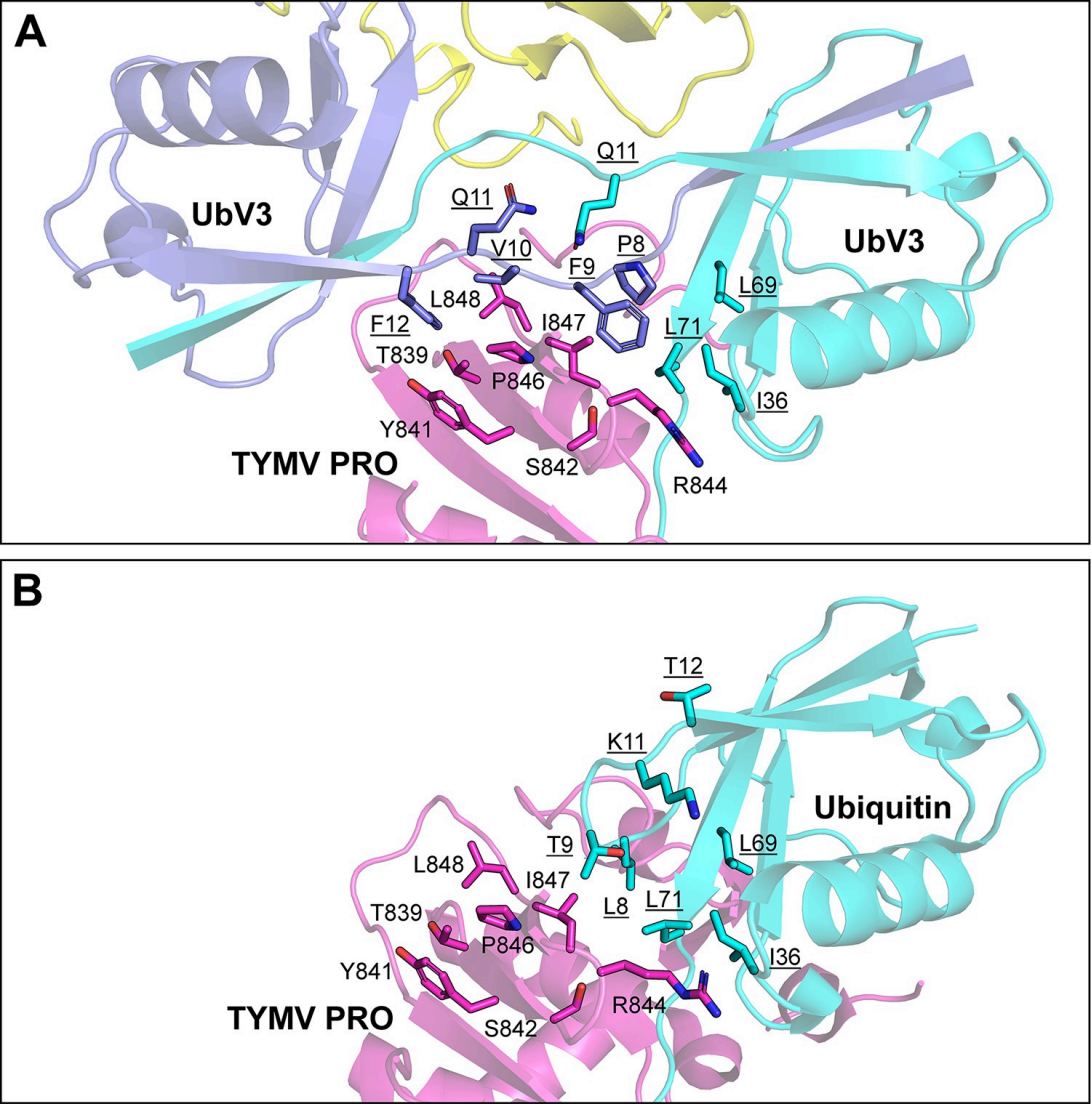
Interactions between TYMV PRO and UbV3 other than its C-terminal tail. TYMV PRO are colored magenta and yellow, UbV3 are colored cyan and blue. Key residues are labeled and underlined for UbV3. (A) Key residues participating in the hydrophobic network of UbV3 and TYMV PRO. (B) Residues of TYMV PRO near the I36 hydrophobic patch of UB (PDB ID: 6YPT). TYMV PRO is colored magenta and Ub is colored cyan.

In addition to the polar contacts described above, the monomers of the UbV3 dimer make extensive hydrophobic contacts to each copy of PRO to which they are bound, which is enabled by the dimerization of UbV3 via the G10V substitution. With some substitutions in UbV3 resulting in additional hydrophobic sidechains, the hydrophobic region near β1-β2 loop (^8^PFVQF^12^) of each monomer of the UbV3 dimer makes extensive packing interactions with complementary hydrophobic regions of the PRO enzymes to which they are bound (Fig 7A). This hydrophobic packing involves the β1-β2 loop (^8^PFVQF^12^) of UbV3, C-terminal I36 hydrophobic patch of another UbV3 chain (I36, L69, L71), and PRO hydrophobic region (aliphatic carbons of T839, Y841, S842, R844, P846, I847 and L848). Similar to UbV3, Ub also interacts with PRO with its I36 hydrophobic patch, as described [26]. However, due to the lack of an extended β1 strand in Ub compared to UbV3, PRO only utilizes a partial surface area for hydrophobic networking with Ub (Fig 7B). As a result, the total binding interface between TYMV PRO and Ub is ~884 Å^2^ according to PISA [46], whereas the interface between PRO and UbV3 dimer is expanded to ~1318 Å^2^ (~961 Å^2^ for a UbV3, which interacts via C-terminal tail, ~356 Å^2^ for another UbV3 which interacts via β1-β2 loop). Thus, the dimeric structure of UbV3 expands the hydrophobic binding interface with PRO compared with Ub, and this likely underlies its high affinity and selectivity for the protease.

### Transgenic expression of UbV3 does not adversely affect *A*. *thaliana* physiology

**Fig 8:**
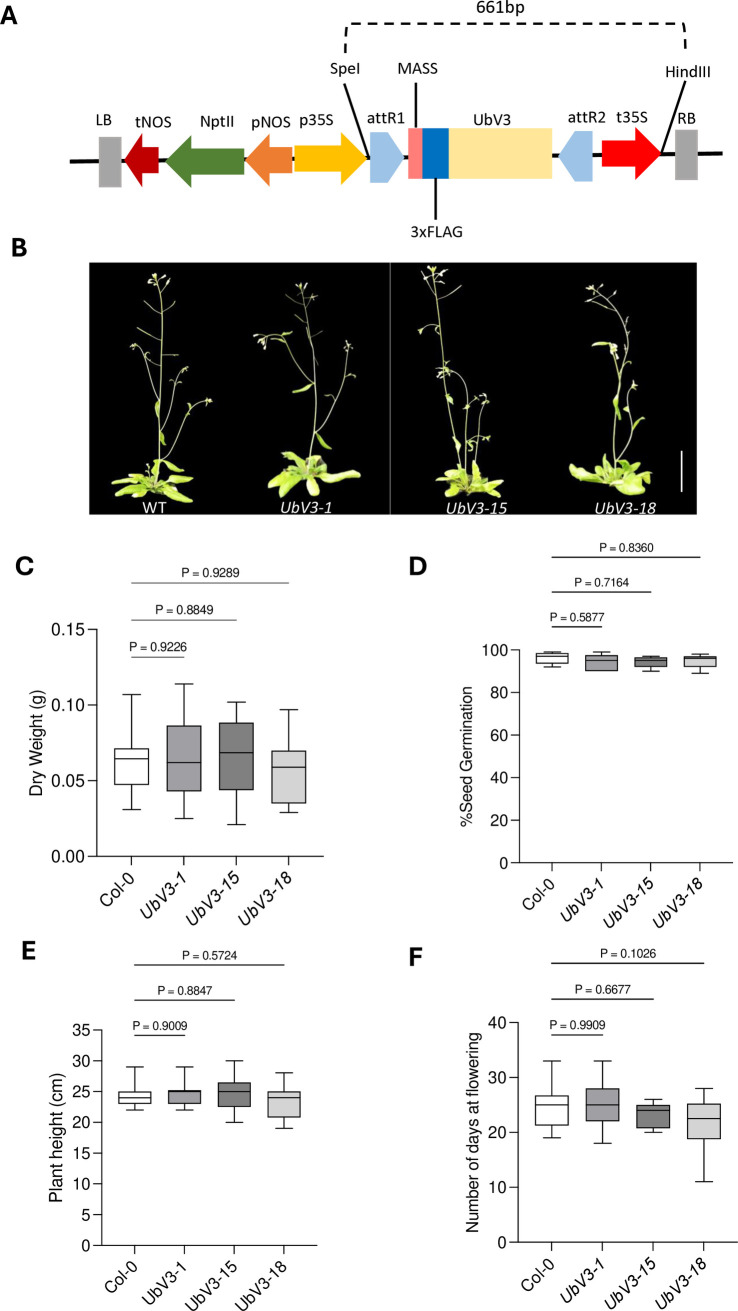
Characterization of UbV3 expression in *A*. *thaliana* lines. (A) T-DNA expression construct pK2GW7 [47]: LB, left border; tNOS, terminator of NptII; NptII, neomycin phosphotransferase II gene; pNOS, promoter of NptII; p35S, CaMV 35S promoter; attR1 sequence; MASS, Met-Ala-Ser-Ser sequence; 3x FLAG tag sequence; UbV3, open reading frame; attR2 sequence; t35S, CaMV terminator sequence and RB, right border. (B) Phenotypic appearance of WT *A*. *thaliana* and the three transgenic lines *UbV3-1*, *UbV3-15*, and *UbV3-18* (scale bar, 30mm). (C) Dry weight after 21 days of seed germination. (D) Seed germination percentage. (E) Plant height. (F) Number of days at flowering. Data in panels C, D, E, and F represent three repeated experiments (Mean ± SD). The dry weight, number of days at flowering, and seed germination percentages of Col-0 and transgenic lines *UbV3-1*, *UbV3-15*, and *UbV3-18* are not significantly different (*p* >0.05).

Having demonstrated the ability of UbV3 to potently and selectively block PRO activity *in vitro*, we next determined if this ability could be used to suppress TYMV replication *in planta*. To explore this possibility, transgenic *A*. *thaliana* lines were developed that constitutively expressed the UbV3 protein via the 35S promoter derived from the CaMV [47,48]. (Fig 8). Following *Agrobacterium*-mediated transformation [49], *A*. *thaliana* transformants were first screened by PCR to detect genomic insertion of the UbV3 expression cassette, followed by Western blot analysis to verify the expression of the UbV3 protein (S12 Fig). Three transgenic plant lines (*UbV3-1*, *UbV3-15* and *UbV3-18*) were confirmed to express UbV3 protein and thus selected for further studies (S12 Fig).

To evaluate whether UbV3 expression affected the phenotype of *A*. *thaliana*, comparative analyses were conducted between the three transgenic *A*. *thaliana* lines expressing UbV3 and the wild-type Col-0 parental line. Phenotypic analyses were conducted on all three transgenic lines (*UbV3-1*, *UbV3-15*, and *UbV3-18)*. Various growth parameters were evaluated, including seed germination, dry weight accumulation, flowering time, and plant height (Fig 8). The results of the phenotypic comparisons revealed no statistically significant differences (p >0.05) between the WT and transgenic lines. The findings suggested that the expression of UbV3 did not affect general plant physiology under the experimental conditions employed, which is consistent with UbV3 being highly selective for PRO (Fig 4).

### UbV3 protein is present in the cytosol and organelle fractions of transgenic *A*. *thaliana*

**Fig 9:**
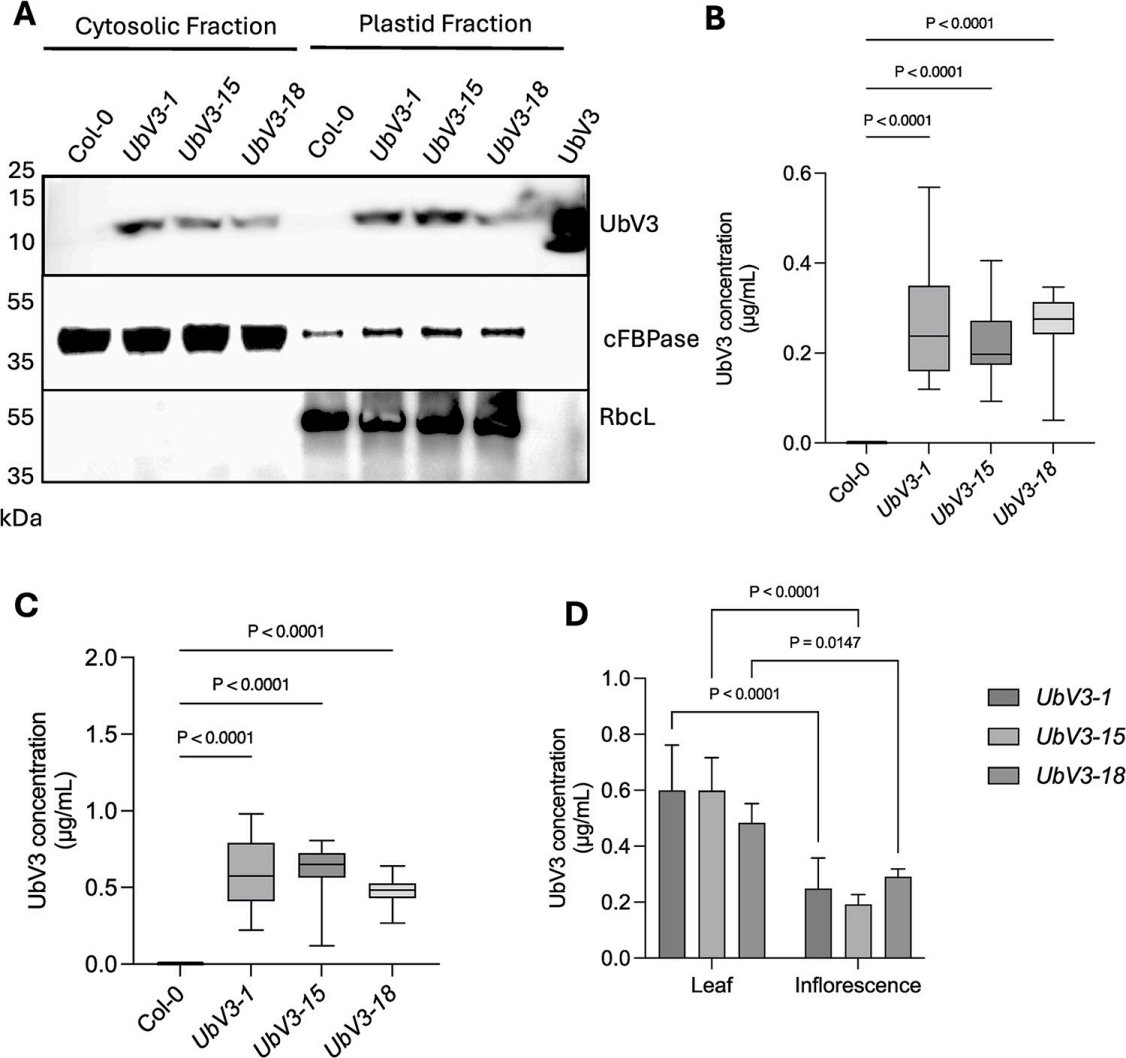
Subcellular fractionation and quantification of UbV3 transgene expression in leaf and inflorescence parts in WT and transgenic *A*. *thaliana*. (A) Western blot analysis of differential centrifugations of protoplast lysates from transgenic *A*. *thaliana*. Lanes 1–4, supernatant fraction at 100,000xg (cytoplasmic fraction); lanes 5–8, pellet fraction (mitochondrial and chloroplast fraction) at 10,000xg; Lane 9, UbV3 protein standard. Western blot analysis of UbV3 detection was performed with monoclonal Anti FLAG M2 as the primary antibody and goat anti-mouse IgA:HRP conjugate as the secondary antibody. Polyclonal antibodies targeted against *Arabidopsis* cFBPase (cytosol) and RbcL (plastids) were applied to the same samples and the goat anti-rabbit IgA:HRP conjugate as the secondary antibody to visualize via western blot analysis. The amount of UbV3 expression in (B) inflorescence and (C) foliage parts was quantified using a FLAG-tag detection ELISA kit. The data were obtained from two repeated experiments. Mean ± SD. (D) the amounts of UbV3 expressed in leaf and inflorescence in transgenic *A*. *thaliana* are significantly different (*p*<0.05).

Since TYMV replication occurs at the periphery of chloroplasts and protein maturation occurs in the cytosol of infected plants [50–52], we carried out subcellular fractionations of protoplasts isolated from leaf tissue of UbV3-expressing *A*. *thaliana* to determine where the UbV3 protein was localizing in plant cells. Cytosolic and plastid fractions from protoplasts (the latter of which is enriched in mitochondria, chloroplasts, and plastids [53] contained UbV3 protein according to Western blot analysis (Fig 9A). This result revealed the presence of UbV3 protein where it would be needed to block PRO activity and suppress TYMV replication in the host cell. We also quantified the amount of UbV3 expressed in inflorescence versus leaf parts of the transgenic lines *UbV3-1*, *UbV3-15* and *UbV3-18* by ELISA and found that UbV3 was expressed primarily in leaf tissue and to a lesser extent in inflorescence (Fig 9B–9D).

Having identified UbV3 protein in the cytosol and plastid fractions of transgenic *A*.*thaliana* lines, we wanted to confirm if the expressed protein was functional and able to inhibit PRO activity. Indeed, we found that cytosolic extracts from transgenic *A*. *thaliana* contained functional UbV3, as these extracts inhibited purified recombinant PRO in a concentration-dependent manner, whereas cytosolic extracts from the parent Col-0 *A*.*thaliana* did not (S13 Fig). Taken together, our structural and functional studies of UbV3 suggested that the protein was well-suited to inhibit PRO in plant cells and consequently suppress TYMV replication *in vivo*.

### UbV3 suppresses TMYV replication in planta

**Fig 10:**
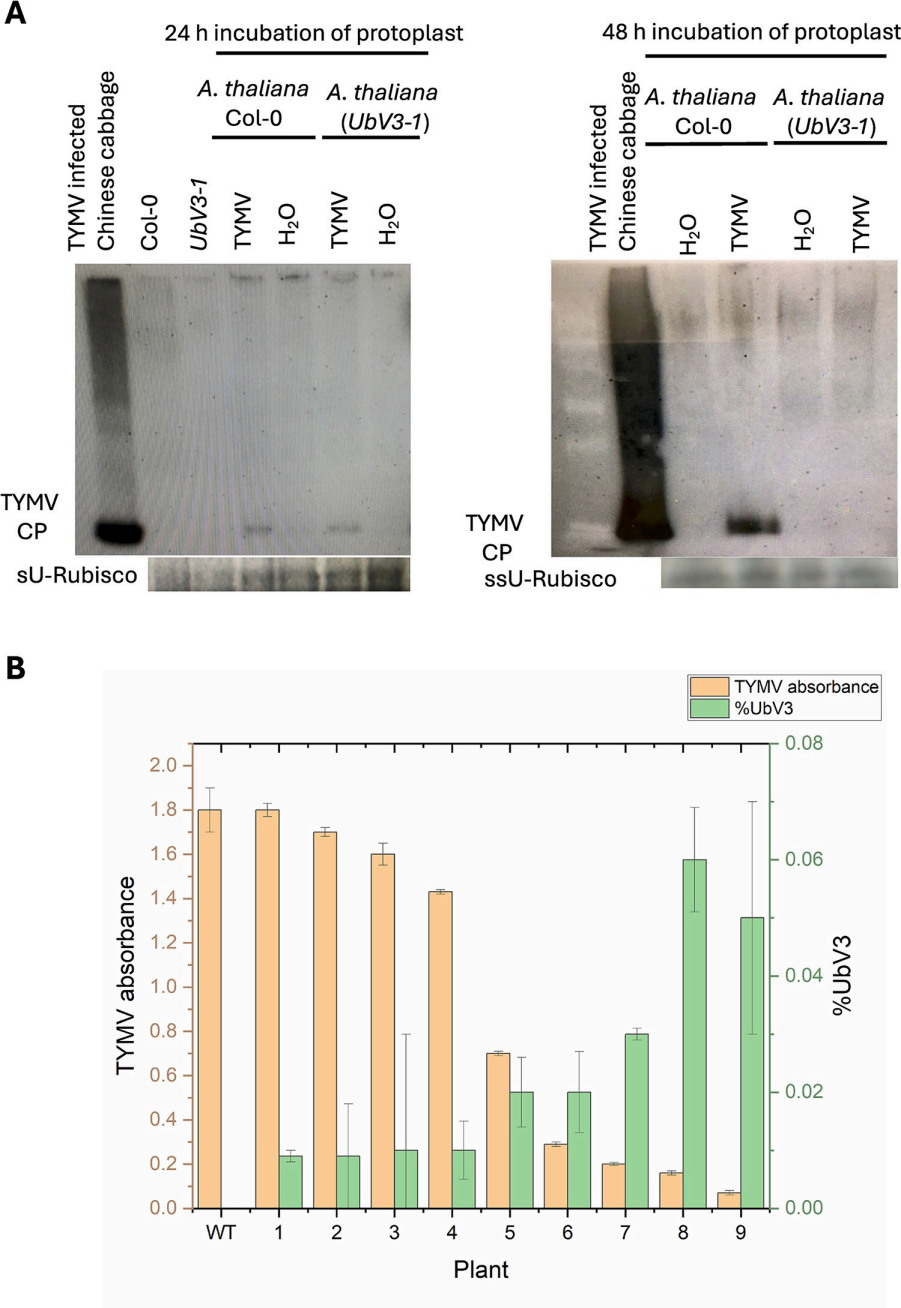
UbV3-mediated suppression of TYMV replication *in planta*. (A) Protoplast infection study after 24-hour (left) and 48-hour (right) incubation with TYMV. Western blots were probed with anti-TYMV antiserum, and coat protein was detected under chemiluminescence. The loading control of a small subunit of Rubisco (ssU-Rubisco) was shown as a protein loading control. (B) Detection of TYMV coat protein accumulation versus UbV3 expression level in the transgenic *A*. *thaliana UbV3-1* line and parent Col-0 plants. The accumulation of TYMV was detected using ELISA, and the expression of UbV3 was quantified using a FLAG-tag detection ELISA kit. The data were statistically analyzed using Pearson correlation analysis, and a strong negative correlation was observed in the Transgenic line *UbV3-1*(Pearson r = -0.8).

To gain insight into the ability of UbV3 to suppress cellular replication of infectious TYMV, protoplasts prepared from leaves of transgenic *A*. *thaliana UbV3-1* and the parent Col-0 line were infected with TYMV for 24 to 48 hours, followed by western blot analysis to detect the presence of TYMV coat protein (Fig 10A). Protoplasts were primarily comprised of mesophyll cells, which are the main photosynthetic cells of the plant leaf. After a 24-hour incubation in the presence of infectious TYMV RNA, the accumulation of TYMV coat protein was similar in transgenic protoplasts and wild-type Col-0. However, after 48-hour incubation, there was no longer any detectable coat protein in transgenic protoplasts, whereas coat protein continued to accumulate in the parent Col-0 protoplasts. This remarkable reduction in viral coat protein suggested that UbV3 interfered TYMV replication, where the lag phase may be due to the response time needed for the Ub-proteosome (UPS) system to clear viral protein from the protoplast generated directly off the initial infectious RNA transcripts.

To assess whether UbV3 expression could suppress TYMV replication in whole plants, we inoculated transgenic *A*. *thaliana UbV3-1* and wild-type Col-0 plants with infectious TYMV particles using an established foliar spray method [54]. The infection was performed at two intervals before and after the bolting of *A*. *thaliana* plants using the foliage spray technique. The efficacy of the method was initially verified using wild-type Col-0, where the presence of TYMV genomic RNA was detected in plants sprayed with infectious TYMV particles (S14 Fig). The difference in TYMV viral load between transgenic and WT plants was analyzed by determining the presence of TYMV coat protein in bulked leaves and inflorescences of inoculated plants using a commercially available TYMV ELISA kit, and the expression level of UbV3 in infected plants was determined using a FLAG-tag detection ELISA kit. We found that UbV3 expression levels varied between individual transgenic *A*. *thaliana UbV3-1* plants. Interestingly, transgenic plants expressing higher levels of UbV3 protein had the lowest levels of detectible TYMV coat protein. This negative correlation (Pearson r = -0.8123) between the expression level of UbV3 and TYMV viral load in transgenic *A*. *thaliana UbV3-1* demonstrated that the UbV3 protein was able to suppress TYMV replication *in planta* (S15 Fig). Though the presence of UbV3 was not able to completely clear the TYMV infection, as TYMV proteins were detected in all infected transgenic plants, this may be due to insufficient levels of UbV3 expression in the transgenic *UbV3-1* line that was used for this proof-of-principal study. Future transformation and breeding experiments aimed at increasing the expression level of UbV3 could potentially generate transgenic lines of *Brassica* species that are highly resistant to TYMV infection. The UbV inhibitor strategy could also be used to protect additional economically important plants from infection by Tymoviruses, as well as additional plant viruses that depend on proteases with DUB activity.

## Discussion

To explore the possibility of using protein-based strategies to protect plants from viral infection, we used an engineered variant of Ub to inhibit the activity of the viral protease PRO that is essential to the replication of the model plant virus TYMV. Together with the TYMV-susceptible model plant *A*. *thaliana*, we used this pathosystem to evaluate the antiviral potential of the UbV inhibitor strategy both *in vitro* and *in vivo*.

Having previously identified UbVs that selectively and potently inhibit vDUB from animal viruses, including SARS-1 and 2 and Crimean Congo Haemorrhagic Fever Virus [22,23], we wanted to demonstrate the utility of the approach to block susceptible vDUBs from plant viruses of agricultural importance, of which there are several [26,55–57]. Though we have found UbVs straightforward to identify from phage-displayed libraries when using stable recombinant vDUBs as bait, their ultimate delivery to the cytosol of virus-infected animal cells remains a critical challenge to be overcome before they can be employed as antiviral therapeutics. Aside from recombinant lysosomal proteins that can be successfully sequestered into the lysosome of animal cells via mannose-6-phosphate receptor-mediated endocytosis to treat lysosomal storage disorders [58], mechanisms for intracellular delivery of recombinant proteins remain experimental [59]. In contrast, transgenic expression of cytosolic proteins in plants is a proven technology that overcomes the challenges of exogenous protein delivery. Since UbVs are a protein-based antiviral strategy, we investigated if a UbV could protect plants from viral infection to provide a proof-of-principle example that the approach could reduce losses from viruses in agriculturally important plant species.

UbV3 was identified as a selective and potent inhibitor of PRO (Figs 1 and 4). UbV3 blocked polyprotein cleavage activity (Fig 3) that is essential for TYMV replication [27], and also, DUB activity that usurps cellular Ub-dependent antiviral mechanisms [14,15]. Notably, PRO has been found to deubiquitinate the RNA-dependent RNA polymerase (RdRp) of the TYMV replicase complex, thereby rescuing the polymerase from K48-polyubiquitin-mediated proteasomal degradation [14,15]. The DUB activity of PRO thus appears to decrease RdRp turnover to enhance viral replication in plant cells [14,15]. We found that this activity is inhibited with increasing concentrations of low micromolar amounts of UbV3 (Figs 1–3). Together, we found that UbV3 could be used to block polyprotein cleavage and DUB activity, which was expected given that the UbV3 dimer occupied the active site and the majority of the Ub recognition site of PRO, according to our structural studies of the complex formed by the proteins.

Based on structural analysis, we found that UbV3 forms a dimer (Fig 5A) that is facilitated by a G10V substitution. Dimerization resulting from a G10V substitution has previously been found to enhance the affinity of a UbV for a specific Ub interacting motif [33]. This is consistent with the effect of this substitution in the UbV3 dimer described here, since reverting V10 back to glycine disrupts dimerization and dramatically reduces the binding affinity of UbV3-(V10G) for the protease (Fig 6). Dimerization accompanied by the loss of the β-turn may lead to structural instability of the UbV3 molecule, however, additional hydrophobic interactions between two UbV3 molecules involving P8 and F9 appear to compensate for this loss, as described above (Fig 5F).

The X-ray crystal structure of the covalently linked PRO-UbV3 complex agrees with the SAXS *ab initio* envelope model of the non-covalent complex (Fig 6A), and this suggests that the binding conformation is not disturbed by the introduction of a covalent bond that was needed to crystallize the complex. Further, given the extensive interactions of the L71–R74 region of UbV3 within the active site cleft of PRO (Fig 6), it also suggests that the covalent linkage of G75 to the active site cysteine of PRO does not significantly affect the natural binding mode of UbV3 to the protease. Finally, no significant interactions exist between the two PRO chains of the asymmetric unit of the crystal structure, which indicates that the complex is primarily held together by the interactions between UbV3 and PRO.

Compared with WT Ub, UbV3 dimerization creates a larger hydrophobic binding interface with PRO, where hydrophobic sidechains on this β1-β2 loop (F9, V10, F12) appear to form an extended hydrophobic network with PRO, thereby increasing affinity (Fig 7A). Utilizing this extended binding interface and its structural flexibility, the UbV3 dimer experiences conformational change when it binds to PRO, especially in the β1-β2 region where most of the substitutions reside (S8 Fig). In summary, dimerization of UbV3 may not be caused by sole amino acid substitution, but rather by multiple substitutions acting together. Substitutions made additional binding interface for PRO/DUB, which includes an extended hydrophobic network. Further substitutions on other sites on UbV3 may promote its efficiency of inhibition.

A previously determined X-ray crystal structure of PRO bound to WT Ub suggested that although K6 of Ub was involved in crystal packing contacts with a neighbouring molecule, it might participate in binding PRO in solution by forming a salt bridge with E759 of the protease [26]. However, a molecular dynamics simulation of the complex did not support this hypothesis due to distance constraints [26]. Interestingly, K6 is substituted to R6 in UbV3, and the additional length provided by the arginine sidechain enables a convincing ~3.3 Å salt bridge to form with the side chain of E759 (Fig 6D). Reverting the R6 substitution back to lysine or to alanine resulted in a measurable reduction in the ability of UbV3 to block PRO activity (S9 Fig). Given the previous observation that K6 of Ub was not close enough to engage productively with E759 of PRO, it was interesting that a UbV was selected from a phage-displayed library with a substitution that closed the distance by using the guanidinium group of Arg, the only amino acid able to form an adequate salt bridge.

The hydrophobic I44 patch on Ub is commonly found to participate in interactions with other proteins [43]. Surprisingly, the I44 patch of UbV3 (I44, L8, H68, V70) does not engage with PRO. L8 was substituted to proline, which instead participates in a hydrophobic network extended from the I36 patch interface on PRO (Fig 7A). The region of PRO adjacent to the hydrophobic I44 patch of UbV3 primarily contains residues with polar sidechains (S10 Fig). Thus, it is curious that UbVs with polar substitutions in the I44 patch were not selected by phage display, as it is conceivable that such substitutions could contribute to increased binding affinity between the proteins. Nonetheless, UbV3 is a potent inhibitor of PRO, and its low nanomolar binding affinity is similar to those of UbV inhibitors we have identified for vDUBs from other viruses [22,23].

TYMV infection typically induces characteristic symptoms in the infected plants, such as mosaic patterns of light and dark green patches on leaves, yellowing, stunting, leaf distortion, deformed flowers and reduction in plant vigour [29]. This results because the viral replication complex (VRC) is associated with the chloroplast envelope, particularly promoting the formation of peripheral vesicles and cytoplasmic invaginations in the lipid bilayer of the chloroplast membrane [60]. Our finding that UbV3 was expressed in the cytosolic and plastids (chloroplast/mitochondria) of transgenic *A*. *thaliana* suggested that it would be available to co-localize with TYMV replication complexes that form at the periphery of chloroplasts [50–52], thereby blocking PRO and reducing TYMV replication. This was supported by our subsequent infection study analysis (Fig 10).

To assess whether UbV3 affects TYMV accumulation *in vivo*, *UbV3-1* transgenic line and wild-type *A*. *thaliana* Col-0 plants or protoplasts were subjected to TYMV infection. TYMV infection was quantified by the amount of viral coat protein present in the infected plants and the detection of coat protein in the protoplasts. Results from the protoplast infection study revealed that TYMV accumulation was reduced after 48 hours of incubation. However, we observed more variable results from our whole plant infection studies, possibly because TYMV does not infect *A*. *thaliana* aggressively. Also, TYMV replicates either a low extent or a great extent in different tissues. Moreover, the level of UbV3 expression was different between transgenic plants (Fig 9), possibly due to transgene copy number, site of the insertion in the plant genome, epigenetic mechanisms, and repressor proteins in the plant [61–64]. Therefore, the protoplast infection data of the virus decline in mesophyll protoplasts at 48 hours was not a dynamic process in a multicellular plant, and we need to optimize the delivery of UbVs into targeted cellular compartments.

Despite this variability, we observed coat protein reduction that was negatively correlated (Pearson’s r = -0.8) to the level of UbV3 expression. The strong correlation demonstrated that higher levels of UbV3 expression were more effective at suppressing TYMV replication in plants. Thus, future efforts to maximize UbV expression, possibly targeting the UbV3 protein to the outer chloroplast membrane and confirming its expression in crucial cell types and tissues that support TYMV replication and movement, could enhance the efficacy of this antiviral approach.

Protein-based inhibitors may provide a long-lived infection control strategy [65–67] without affecting plant growth and specifically targeting viral replication in the plants. Overall, they offer the potential for long-lasting protection compatible with plant growth, and more research is needed to identify the plant-virus interaction and delivery of these protein-based inhibitors to target plant tissues.

## Materials and methodology

### Construction of protein expression plasmids

Synthetic DNA (IDT: Integrated DNA Technologies) coding for the catalytic domain of the TYMV PRO and the catalytic domain of AtAMSH3 [38] was amplified by PCR using primers listed in S2 Table and ligated into pGEX-6P-1 (GE Healthcare) using BamHI and XhoI restriction sites (5^’^ and 3^’^, respectively). Synthetic DNA coding for the catalytic domain of TYMV PRO, AtUCH2, AtOTLD1 and AtAT3G was amplified by PCR using primers listed in S2 Table and ligated into pET19b (Novagen) and pET28a using NdeI and BamHI restriction sites.

### Selection of ubiquitin variants

The phage-display technique was developed for the ubiquitin variants as previously described [21]. Protein immobilization and UbV selections were made according to established protocols [22,23,68]. Briefly, purified viral protease (GST-tagged PRO) was coated on 96-well MaxiSorp plates (Thermo Scientific 12565135) by adding 100 μL of 1 μM proteins and incubating overnight at 4°C followed by five rounds of selections using the phage-displayed UbV library as described [22,23]. After the fifth round of binding selections, phages with improved binding affinity were identified by ELISA and their DNA sequences were obtained [68,69].

### Construction of mutants of UbV3 using site-directed mutagenesis

Single point mutations (V10G, R6K, R6A) were generated in the UbV3 expression construct pET53 DEST-UbV3 using the Q5 site-directed mutagenesis kit (NEB, cat. E0554S). *E*. *coli* BL21(DE3) Gold cells were transformed with a mutated plasmid.

### Protein expression and purification of PRO and plant DUBs

Expression plasmids described above were used to transform *Escherichia coli* BL21 (DE3) GOLD cells (Stratagene) for subsequent recombinant protein production. Transformed *E*. *coli* was grown overnight at 37°C in LB (lysogeny broth) containing appropriate antibiotics (Ampicillin,150mg/mL or Kanamycin, 50mg/mL). The overnight culture was then used to inoculate 1 l of fresh LB with appropriate antibiotics (1:50 dilution) and was grown at 37°C with shaking to an OD_600_ of 0.7 to 0.8. Expression of GST-tagged enzymes (from pGEX-6P-1 constructs) or His6-tagged enzymes (from pET19b and pET28a) was induced by the addition of 0.5 mM IPTG and then incubated with shaking at 16°C for an additional 18 h. After 18 hours of incubation, cells were pelleted by centrifugation.

Cell pellets of GST-tagged enzymes were resuspended in ice-cold lysis buffer (50 mM TRIS- HCl pH 8.0, 300 mM NaCl and two mM DTT) and lysed using an Avestin Emulsiflex C3 high-pressure cell homogenizer. Cell lysates were then clarified by centrifugation. The supernatant containing GST-tagged enzymes was mixed end-over-end for 1 h at 4°C with GST-Bind resin (Millipore), pre-equilibrated in the lysis buffer. The lysate/resin slurry was poured into a gravity column and washed with 2 column volumes of the lysis buffer, followed by elution of the GST-tagged fusion proteins with the lysis buffer supplemented with 10 mM reduced glutathione (pH 8.0 with NaOH). The GST tag was removed from each enzyme using GST-tagged HRV 3C PreScission Protease (GE Healthcare), which was incubated with the eluted fusion protein in dialysis tubing overnight at 4°C with a two-litre of dialysis buffer (20 mM Tris HCl, pH 8.0, 150 mM NaCl, and 2 mM DTT). After dialysis, the enzymes were purified using a Superdex 75 (GE Healthcare) gel filtration column and Superdex 200 (GE Healthcare) gel filtration column.

Cell pellets of His_6_-tagged enzymes were resuspended in ice-cold lysis buffer (50 mM Tris HCl, pH 8.0, 300 mM NaCl, 2 mM DTT, and 5 mM imidazole) and lysed similarly to the GST-tagged enzymes. Lysates were then clarified by centrifugation 22,000rcf, and supernatants containing proteins were mixed end-over-end for 1 h at 4°C with nickel-nitrilotriacetic acid resin (Qiagen), pre-equilibrated in the lysis buffer. The mixture of lysate and resin slurry was then poured into a gravity column and washed with 20 column volumes of the lysis buffer, followed by 10 column volumes of the lysis buffer supplemented with 15 mM imidazole, followed by 10 column volumes of the lysis buffer supplemented with 30 mM imidazole and finally eluted with the lysis buffer supplemented with 450 mM imidazole. The eluted proteins were dialyzed against 2 litres of dialysis/gel filtration buffer (20 mM TRIS-HCl pH 8.0, 150 mM NaCl and 2 mM DTT) overnight at 4°C and then further purified using a Superdex 75 gel filtration column and Superdex 200 gel filtration column.

### Expression and purification of UbVs

Expression plasmids containing UbV sequences from phage display selections (pET53-DEST-UbV1, pET53 DEST-UbV2, pET53 DEST-UbV3) as well as mutants of UbV3, were used to transform *E*. *coli* BL21 (DE3) Gold cells for subsequent expression of His6-FLAG-tagged UbV proteins. Purification of the expressed UbV proteins was carried similarly to PRO and plant DUBs described above.

### ELISA assays to evaluate binding specificities

To measure the half-maximal binding concentration (EC_50_) of UbVs binding to viral proteases, the concentration of UbVs or wild-type Ub was varied from 0 to 4 μM (24 points, 1:2 dilution), while the concentration of target proteins immobilized on the plate remained at 1 μM. EC_50_ values were calculated using the GraphPad Prism software with the built-in equation formula (non-linear regression curve). Three potential UbVs (UbV1-3) were identified.

The ELISA assay was used to determine the binding specificities of the UbVs towards a panel of plant DUBs and PRO. As previously described [22], proteins were immobilized on a MultiScreen 96-well polystyrene flat bottom clear ELISA high binding plate (Millipore) at 2 μg/mL and incubated overnight at 4°C. Then, 300 nM of FLAG-tagged UbVs was added and bound UbV protein was detected by the addition of an anti-FLAG HRP antibody (GenScript). The colourometric assay was initiated by the addition of the TMB (3’, 5, 5’ tetramethyl benzidine) peroxidase substrate (mixture of equal volume of TMB substrate A and B, BioLegend). The reaction was stopped by adding a BioLegend Stop Solution (Cat. No. 423001), turning the colour from blue to yellow. The absorbance of the plate was read at 450 nm using SpectraMax iD5 Multi-Mode Microplate Reader (Molecular Device, USA), and the mean of the absorbance was represented in a heat map on a white (minimum value)–black (maximum value) gradient using GraphPad Prism software (v.9.5.1).

The initial rates of each reaction were calculated to measure the IC_50_ values. Assuming the enzymes follow Michaelis-Menten kinetics, the initial velocity (intensity) was plotted against UbV3 concentrations using GraphPad Prism. The data was fitted to a four-parameter sigmoid Hill curve model (Sigmoidal, 4PL) [70], and IC_50_ values were calculated using GraphPad Prism [71]. The hydrolysis of Ub-AMC was measured at 25°C Excitation/Emission 360/460nm, Low PMT and 1OD attenuation.

### Gel filtration protein coupling of PRO and UbV3

Purified PRO was mixed with a 2-fold molar excess of UbV3 at room temperature for 1 h, with the addition of TCEP [Tris (2-carboxyethyl) phosphine hydrochloride] to a final concentration of 5mM in order to stabilize the enzyme reaction. To verify protein-protein interactions and purify the resulting complexes, the PRO:UbV3 complex was separated from excess UbV3 by gel filtration. The resulting complex mixture was purified using a Superdex 75 gel filtration column (GE Healthcare), eluting in 20 mM Tris pH 8.0, 150 mM NaCl and 2 mM DTT.

### Deubiquitinase activity assays

All deubiquitinases, including PRO, were assayed against the fluorogenic substrate analogue 7-amino-4-methyl-coumarin (AMC)–Ub (Boston Biochem). The reaction buffer for all assays consisted of 50 mM Tris-HCl (pH 8.0), 150 mM NaCl, and 2 mM DTT. The substrate in the reaction buffer was placed in a black, flat-bottom 96-well microplate (Corning Life Sciences), and the different concentrations of the enzyme were added immediately before readings [72]. The final reaction volume was 100 μl. Time-course kinetics assays were carried out using a SpectraMax iD5 microplate reader (Molecular Devices). The instrument was set to excitation of 360 nm and emission of 460 nm. The PMT gain was medium, and reads were taken every 20s. The graph of deubiquitinase activity for each deubiquitinase was plotted using the relative fluorescence units (RFU) against time (sec) using GraphPad Prism.

### K48-linked polyUb chain cleavage assay with the presence of UbV3

Using recombinant human polyubiquitin [K48- linked (UC-220), Boston Biochem)] as substrates, the ability of UbV3 to inhibit PRO activity against these substrates was measured in 50 mM Tris-HCl, pH 8.0, 100 mM NaCl, 1 mM DTT containing 1μg of poly-Ub substrate, 2 μM PRO and 0–2 μM UbV3. After incubation at 25°C for 30 min, reactions were stopped by adding 2X Tris-Tricine SDS PAGE sample buffer. Protein samples were denatured at 40°C for 20 minutes. Reactions were visualized by carrying out TRIS-Tricine SDS PAGE (10%) and subsequent detection using a Pierce Silver Stain Kit (Thermo Fisher Scientific).

### Cleavage of the Pro↓Hel site from the TYMV polyprotein by PRO and its inhibition by UbV3

A fusion protein containing the PRO cleavage site Pro↓Hel from the TYMV polyprotein was constructed as follows: The open reading frame (ORF) of PRO and the first 10 amino acids of Hel were fused to the N-terminus of the glycosidase NagZ (GenBank ID: OEOG01000025) [37,73]. The Pro↓Hel cleavage site is located between PRO and Hel (Fig 3A). NagZ was used to replace Hel to ensure expression of a soluble fusion protein. This fusion construct was synthesized by IDT and cloned into pET Duet using AsiSI and XhoI sites. The ORF encoding UbV3 was cloned into the partnering cassette of pET Duet using BamHI and SalI sites. Two pET Duet constructs were created, one with UbV3 and one without (Fig 3A).

The resulting plasmids were used to transform *E*. *coli* BL21(DE3) for subsequent protein expression. Transformed *E*. *coli* were incubated in LB medium with 100 μg/mL of ampicillin at 37°C, 180 rpm. When the OD_600_ reached 0.5, IPTG 0.5 mM was added to the medium. The temperature was lowered to 18°C, and the culture medium was harvested overnight. The cells were lysed by homogenizer in a lysis buffer (20 mM Tris-HCl pH 7.5, 150 mM NaCl, 10mM beta-mercaptoethanol). After centrifugation at 13000rpm, 4°C for 50 minutes, supernatant was collected for analysis.

Protein in clarified lysates were separated on 12% tricine-SDS-PAGE and then transferred to PVDF membrane. The membrane was stained with Ponceau S to verify consist of sample loading (S3 Fig). The membrane was blotted using anti-Flag antibody-HRP(GenScript, A01428), and anti-HIStag antibody-HRP (Thermo, PI15165). The cleavage or inhibition of cleavage of the PRO-NagZ fusion protein was determined by visualizing the cleavage products and intact fusion protein.

### Covalent linkage of UbV3 and PRO

The open reading frame for UbV3 (lacking the N-terminal 6xHis and FLAG tag) was ligated into vector pTYB2 using restriction enzymes *NdeI* and *SmaI* to generate pTYB2-UbV3, from which UbV3 was expressed, purified and modified at its C-terminus using 3-bromopropyl hydrobromide as previously described [45]. The sample was dialyzed in buffer (20mM Tris pH 8.0 150mM NaCl), and then purified by gel filtration chromatography (Superdex 75). 1:1 molar ratio mixture of purified UbV3-Br and PRO was incubated at 37°C for 2 hours. After incubation, it was purified by gel filtration chromatography (Superdex 75) with the same buffer.

### Crystallization and structure determination

A single peak corresponding to the dimer of UbV3 was pooled from the gel filtration chromatography and concentrated to 8 mg/mL for crystallization trials. Initial crystals were observed from preliminary screens (PEGs, PEGs II suite, NeXtal Cat. #130904, #130916) then optimized conditions were set up for sitting drop vapour diffusion crystallization at 20°C. The total drop volume was 2μL, containing equal volumes of the protein and the crystallization solution. Diffraction quality crystals of dimeric UbV3 appeared in 15% PEG 1000, 0.5M Ammonium Sulfate after 7 days. Crystals were cryoprotected with mother liquor supplemented with 15% glycerol and flash cooled in liquid nitrogen. X-ray diffraction data were collected using a Rigaku MicroMax-007 HF X-ray generator and R-axis detector. Data were indexed and integrated by XDS package [74], merged, and scaled by Aimless [75]. The structure was determined by molecular replacement using Phaser [76] and ubiquitin (PDB: 1UBQ) as the search model. Atom coordinates of the exchanged loop β1-β2 region were manually fitted and refined by Phenix.refine [77].

Covalently linked PRO:UbV3 dimer complex was concentrated to 12mg/mL, and crystallized by mixing an equal volume of protein sample and crystallization mother liquor (1.5M ammonium sulphate 100mM Bis-Tris pH 6.5 100mM NaCl) using the sitting drop vapour diffusion method. Crystals appeared 2 weeks after set up, and were cryoprotected in the mother liquor supplemented with 22.5% ethylene glycol before flash cooling in liquid nitrogen. The X-ray data collection strategy was the same as described above. Collected data were indexed and merged using XDS and scaled by Aimless. The structure was determined by molecular replacement using Phaser, using TYMV PRO (PDB: 6YPT) and the UbV3 dimer determined before as search models. The complex was further fitted into the resulting electron density manually and refined by Phenix.refine.

### Small-angle X-ray Scattering (SAXS) analysis

Purified UbV3 dimer from gel filtration chromatography was sent to B21 BioSAXS beamline at Diamond Light Source (Didcot, Oxfordshire, UK). A complex of UbV3 dimer and PRO was purified by gel filtration chromatography before being sent to DLS. All samples were in the same buffer (20mM Tris pH 8.0, 150mM NaCl, 2mM DTT). Samples were concentrated to ~5mg/mL and 80μL of samples were loaded on the Shodex KW402.5-4F column at the beamline B21. The ATSAS suite version 2.8 [78] and Chromixs [79] were used to analyze the scattering data, with Chromixs used to correct for buffer baseline on the samples’ peak intensity. Guinier analysis (q^2^vs. ln(I(q))) [80] was employed to determine the quality of the samples and the radius of gyration (R_g_) (S11 Fig). The folding was characterized by generating a dimensionless Kratky plot (qR_g_ vs. qR_g_^2*I(q)/I(0)) [81]. Real-space R_g_ and the maximum particle dimension (D_max_) were calculated using a paired distribution function (P(r)). The P(r) plot served as input data for DAMMIN, which produced twenty models in each condition using different seeds for each model and equal parameters to ensure symmetry [82]. An average model was obtained using DAMAVER, and a filtered representative structure model was created using DAMFILT [82,83].

### Molecular cloning and construction of UbV3 expressing transgenic plant lines

Synthetic DNA (Integrated DNA Technologies) of expression cassette consisted of the UbV3 open reading frame with a linker between attB1/attB2, a MASS (Met-Ala-Ser-Ser) motif to initiate translation and enhance protein stability [84], a 3x FLAG tag for protein detection, attB2, a linker between attB2 and T35s sequence. The expression cassette was amplified by PCR using primers listed in S2 Table. The resulting PCR amplicon was ligated into a pK2GW7 binary vector using restriction sites SpeI and HindIII to generate an N-terminally FLAG-tagged UbV3 expression cassette for integration into *A*. *thaliana* via *Agrobacterium*-mediated transformation. The recombinant binary vector, pK2GW7-UbV3, was transformed to *Agrobacterium* GV3101::pMP90 strain via electroporation.

Cold stratification-treated sterile *Arabidopsis* seeds were kept at 4°C for two days. Plates were then moved into a plant growth chamber (20–22°C and 50% relative humidity) under a 16h light and 8h darkness cycle. Fluorescent bulbs provided white light (100 μmol m^-2^ sec^-1^). Two-week-old seedlings grown under these conditions were transplanted from plates to soil and grown further under the same lighting and temperature conditions.

*Arabidopsis thaliana* Colombia (Col-0) was used as the wild-type strain for generating all transgenic lines. Transgenic *A*. *thaliana* was generated using *Agrobacterium*-mediated transformation by floral dip and a floral spray with *Agrobacterium tumefaciens* GV3101 [85]. Kanamycin resistance was used to select transgenic plants and zygosity. The presence of the transgene was confirmed by PCR, and expression of UbV3 protein was detected by western blot analysis using an anti-FLAG antibody (BioRad). Three transgenic lines (*UbV3-1*, *UbV3-15* and *UbV3-18*) were subjected to phenotype analysis (Fig 8). The zygosity of these transgenic lines was determined based on kanamycin antibiotic resistance, as the T-DNA binary vector pK2GW7 contains the neomycin phosphotransferase II gene [47].

### Phenotypic analysis

Half-strength Murashige and Skoog (MS) media containing 0.8% phytoagar and 2% sucrose at pH 5.8 was used for seed germination. Plates were placed in a growth chamber (16 h light/ 8 h darkness) and grown under light conditions until they reached 50% of the inflorescence to be produced. Five hundred seeds were used for the seed germination experiment.

Thirty wild-type and transgenic plants were analyzed for flowering time and shoot growth characteristics. Two weeks old, seedlings were transplanted from plates to the soil. For flowering time analysis, the total duration of flowering was measured. The height of the plant and dry mass weight after 21 days of seed germination were analyzed for shoot growth analysis. The data was obtained from three repeated experiments. Experimental data shown in this study are means with 95% confidence intervals (C.I). One-way ANOVA was used for data comparison. p-values of 0.05 or less were considered statistically significant. Graphpad Ver. 9.5.1 was used for data analysis.

### Quantification and detection of FLAG-tagged UbV3

Total protein from 100 mg of foliar tissue (transgenic and wild-type) was isolated using lysis buffer (0.12M Tris-HCl pH6.8, 4% w/v SDS, 10% v/v β-mercaptoethanol and 5% glycerol). The expressed UbV3 was quantified using a FLAG-tag (DYKDDDDK) detection ELISA kit following the protocol of the manufacturer (Cayman Chemical 501560).

Transgenic and wild-type tissue samples homogenized in protein loading dye (50mM Tris-HCl, pH 6.8, 100mM DTT, 2% SDS, 10% glycerol, 0.1gL^-1^ bromophenol blue) were boiled at 95°C for 10 minutes. After vortexing followed by centrifuging, 20 μl of the supernatant was loaded and resolved on 12% SDS-PAGE gels. Proteins were then transferred to nitrocellulose membrane. The membrane was blocked with 5% Tris-Buffered Saline Tween-20 (TBST) skim milk for 1 hour at room temperature and then incubated with primary antibody (monoclonal Anti-FLAG M2) solution overnight at 4°C. The following day, the nitrocellulose membrane was transferred to the secondary antibody, Goat anti-mouse IgA: HRP and incubated for 2 hours at room temperature. Following the addition of the HRP substrate, the chemiluminescent signal was detected.

### Subcellular fractionation of FLAG-tagged UbV3

For subcellular fractionation of FLAG-tagged UbV3, differential centrifugation was performed as described [53]. Protoplasts were isolated using a protoplast isolation kit (bioWorld, USA) and resuspended in homogenization buffer (0.4M sucrose, 3mM EDYA, 50mM Tris-HCl and 2mM DTT) and then disrupted with ~5 strokes of a Potter-Elvehjem homogenizer at 4°C (1:2 ratio of cells to buffer). The homogenate was then centrifuged at 800 x g for 15 min at 4°C, and the supernatant was separated from the cell pellet and further centrifuged at 10,000 x g for 15 min at 4°C. The resulting supernatant, which contains mitochondria and plastids, was again separated from the pellet, and further centrifuged at 100,000 x g for 1 h at 4°C to generate a final supernatant that was the cytosolic fraction. The presence of UbV3 was visualized using FLAG-tagged detection western blot analysis, as described above. Polyclonal antibodies against *Arabidopsis* cFBPase (cytosol) [Thermofisher] and RbcL (plastids) [Cedarlane] were used as loading controls and to determine their enrichment in subcellular fractions and using western blot analysis.

### Inhibition of PRO with cytosolic extracts from transgenic *A*. *thaliana*

The inhibition assay using Ub-AMC (Boston Biochem) as a substrate was performed as described [22]. Experiments were performed in assay buffer (50 mM Tris-HCl (pH 8.0), 150 mM NaCl, and 2 mM DTT) containing 1μM Ub-AMC substrate. The cytoplasmic fraction (~10-15kDa) was isolated using ultracentrifugation and centrifugal filtering techniques. The varying concentrations of cytoplasmic fractions of transgenic and wild-type *A*. *thaliana* were tested against 500 nM PRO. Inhibition activity was measured by monitoring the AMC fluorescence emission at 460 nm (excitation at 360 nm) for 30 minutes using a SpectraMax iD5.

### Protoplast and whole plant infection study

For the inoculation of *A*. *thaliana* protoplasts, plants were grown at 22°C under fluorescent lighting with a 12 h day length to suppress bolting. Protoplasts were obtained using a protocol for Chinese cabbage [86] and inoculated using electroporation [9] of synthetic, capped RNA transcribed from plasmid pTYMC [86]. The washed protoplasts were transferred aseptically to the incubation medium (0.55M mannitol-MES pH 5.6 containing Muriashige and Skoog basal medium with vitamins). After 24 h and 48 h of incubation period, protoplasts were collected by centrifugation and frozen at -80C. The infected protoplasts were disrupted to extract the total protein content. The western blot analysis was performed to detect the TYMV coat protein as previously described [86]. The small subunit of Rubisco (ssU-Rubisco) was used as the protein loading control.

Transgenic and wild-type Col-0 *A*. *thaliana* plants were grown at 19–21°C under a 16h light/8h dark photoperiod in a growth chamber, and plants were inoculated with TYMV before the occurrence of floral transition using the foliar spray technique. A virus-containing suspension prepared from freeze-dried leaves of TYMV infected Chinese cabbage in 0.1M NaPO_4_ pH 7.0 was used as inoculum. After the inoculation, plants were allowed to grow in the same conditions, harvested at 28 of post-inoculation. The samples were frozen at -80°C for further analysis. As previously described, the amount of UbV3 expressed in each infected transgenic plant was quantified using a FLAG-tag detection ELISA kit (Cayman Chemical 501560), and the accumulation of TYMV was determined using ELISA (TYMV Detection kit, Creative Diagnostics). Ten wild-type plants were used as a negative control. Detection of TYMV coat protein in whole plants was carried out by ELISA according to the kit manufacturer (Kit# RT-0125; DSMZ-German Collection of Microorganisms and Cell Cultures GmbH, Braunschweig, Germany).

Experimental data shown in this study are means with 95% confidence intervals (C.I). Pearson’s correlation was used for data comparison, and probabilities of 0.05 or less were statistically significant. The Graphpad software Ver. 9.5.1 was used to analyze the data.

## Supporting information

S1 Fig Purification of GST-TYMV PRO and His_6_-TYMV PRO. (A) Chromatogram (FPLC) of His_6_-TYMV PRO using Superdex 75 column (GE Healthcare) equilibrated with 20 mM TRIS, 150 mM NaCl, 2 mM DTT, pH 8.0. The third peak (~78 ml) is TYMV PRO. (B) 12% SDS PAGE gel of purified TYMV PRO without GST tag (cleaved by HRV3c Precision Protease) and the fractions collected from each peak as follows, 1: GST tagged TYMV PRO (~43kDa), 2:GST (25kDa) and 3: TYMV PRO (~18kDa). (C) Chromatogram of His_6_-TYMV PRO. (D) 12% SDS PAGE gel of purified His_6_-TYMV PRO (~18kDa).
(TIFF)

S2 Fig The deubiquitinase activity of TYMV PRO and *A*. *thaliana* plant DUBs. The DUB activity of TYMV PRO (A), AtOTLD1 (B), AtUCH2 (C), AtAMSH3 (D) and AtAT3G (E). The concentrations of enzymes are as follows: dark blue, 1μM; red, 0.5 μM; magenta, 5.0μM; orange, 2.5μM. The black line represents the control (Ub-AMC substrate, 1μM).
(TIFF)

S3 Fig Western blot analysis of UbV3 inhibition of *Pro↓Hel* cleavage. Ponceau S staining of the blots (*above*) is shown to demonstrate and determine equal protein loading (60 μg). Due to the low amount of protein expressed, the location of TYMV PRO-NagZ on the Ponceau stained gels are estimated based on the positions revealed by the Western blot (*below*). Cell lysates were analyzed by Western blotting using anti-FLAG tag antibody(A), and anti-Histag antibody (B).
(TIFF)

S4 Fig Ramachandran plot showing dihedral angles of amino acids on b1-b2 loop (^7^TLTGK^11^). Amino acids of Ub are indicated as red triangle marker (PDB ID: 1UBQ), amino acids of UbV3 were indicated as yellow dot marker.
(TIFF)

S5 Fig Size exclusion chromatography profile of TYMV PRO:UbV3 dimer complex and covalent complex of TYMV PRO:UbV3 dimer. (TIFF)

S6 Fig Alignment of TYMV PRO: ubiquitin complex structure (PDB ID: 6YPT) and TYMV PRO: UbV3. 1:1 complex of 6YPT was superimposed on the half of 2:2 complex of TYMV PRO: UbV3.
(TIFF)

S7 Fig Polar interactions between UbV3 and TYMV PRO other than the active site of TYMV PRO. (A) Salt-bridge between UbV3 R6 and TYMV PRO E759. (B) Hydrogen bond between the backbone amide N atom of UbV3 V10 and the carbonyl O atom of TYMV PRO P846. (C) Hydrogen bond between UbV3 H68 and TYMV PRO N760. Possible interactions are indicated by a dashed line with distance. Residues of UbV3 are labeled with underline, and the key residues of TYMV PRO/DUB active site are labelled.
(TIFF)

S8 Fig Alignment of UbV3 dimers from two different crystal structures. Free UbV3 dimer is colored red, TYMV PRO bound UbV3 dimer is colored green. Single chains of ubiquitin are aligned together to show the orientation of the second ubiquitin chain.
(TIFF)

S9 Fig TYMV PRO Ub-AMC hydrolysis assay with three inhibitor constructs: UbV3, UbV3 R6K and R6A mutants. (TIFF)

S10 Fig TYMV PRO and UbV3 dimer complex structure. TYMV PRO does not form hydrophobic interaction with the I44 patch of Ub. The two copies of TYMV PRO are coloured magenta and yellow and the monomers of the UbV3 dimer are colored cyan and blue. Some key residues are labelled with residue numbers, underlined for UbV3.
(TIFF)

S11 Fig Small Angle X-Ray Scattering (SAXS) data of recombinant TYMV PRO. A) Combined scattering data for TYMV PRO showing the relationship between scattering intensity and scattering angle (q = 4πsinϴ/λ). B) Guinier plots illustrate the calculation of Rg and homogeneity obtained from the low-angle area. C) Dimensionless Kratky plots depicting a globular structure as a result of the Gaussian shape of the curve. D) Normalized pair-distance distribution plots for maximal particle dimension (Dmax) determination from the entire SAXS dataset.
(TIFF)

S12 Fig Selection scheme for *UbV3* transgenic plants. Transgenic plants were generated by *Agrobacterium*-mediated transformation, and plants that showed kanamycin resistance were further screened via PCR and Western blot analysis for the presence of the UbV expression cassette and protein, respectively. The size of the expression cassette (EC) is 661bp. During the initial screening, 20 plants (A: 1–11 plants and B: 12–20) were screened by PCR, and the resulting amplicons were highlighted by blue arrows (M: DNA molecular weight marker). Selected transgenic lines were screened by Western blot analysis to confirm the presence of FLAG-tagged- UbV3 protein (~12kDa) [L: Molecular weight marker]. Three transgenic lines (*UbV3-1*, *UbV3-15* and *UbV3-18*) expressed FLAG-tagged UbV3 (C and D). These lines were allowed to self-pollinate. The *UbV3-1*, *UbV3-15* and *UbV3-18* plants were selected for phenotypic analysis and subcellular fractionation, and the *UbV3-1* line was used for the *in vivo* infection study (After two successive selfings, the only *UbV3-1* transgenic plant lines yielded a stable phenotype (third-generation plants, T3), and all T3 plants expressed UbV3 inhibitor).
(TIFF)

S13 Fig Hydrolysis of 1 mM Ub-AMC by 500 nM TYMV PRO in the presence of varying concentrations of protoplast cytoplasmic fractions. (A) WT *A*. *thaliana* protoplast cytosolic fraction and (B-D) transgenic *A*. *thaliana* protoplast cytosolic fraction from (B) *UbV3-1* (B), (C) *UbV3-15*, and (D) *UbV3-18*. The line symbols represent the concentration of the cytoplasmic fraction (μg), and the black circle line represents the 1 μM of Ub-AMC control.
(TIFF)

S14 Fig Phenotypic changes after TYMV infection and symptom variability. Wild-type *A*. *thaliana* Col-0 (A: H_2_O treatment and B: TYMV infection). (C) Gel electrophoresis of total nucleic acid extracts from wild-type *A*. *thaliana* with and without TYMV infection. The rRNA and chloroplast rRNA are reduced after TYMV infection. (D) TYMV infection experiment setup and symptom variability. The WT (*A*. *thaliana* Col-0) and Transgenic *A*. *thaliana* (UbV3-1) with different generations (T1, T2 and T3) were sprayed with TYMV before flowering and at a late stage (foliar spray). This figure shows the late-stage infection symptoms in plants. Plants were kept under fluorescent lights at ~24°C and 12 h day length. The third transgenic generation plants (T3) were used for the coat protein determination experiment.
(TIFF)

S15 Fig Pearson correlation analysis between TYMV detection and the amount of UbV3 expression in transgenic plants. The data were statistically analyzed using Pearson correlation analysis, and a strong negative correlation was observed in the Transgenic line *UbV3-1*(Pearson r = -0.8).
(TIFF)

S1 Table Data collection and refinement statistics for UbV3 dimer and TYMV PRO-UbV3 complex structures. (TIFF)

S2 Table Primers used to construct the recombinant plasmids. (TIFF)

S3 Table Non-linear regression analysis results of fitting the kinetic profiles of Ub-AMC hydrolysis by TYMV PRO and *A*. *thaliana* DUBs in the presence of different concentrations of UbV3 to equation y = y_0_+ae^bx^.
(XLSX)
