# Supplementary figures and images for: Suppressing Tymovirus replication in plants using a variant of ubiquitin
Source: PLoS Pathog. 2025 Jan 27;21(1):e1012899. doi: 10.1371/journal.ppat.1012899 (PMC11819560; doi:10.1371/journal.ppat.1012899)

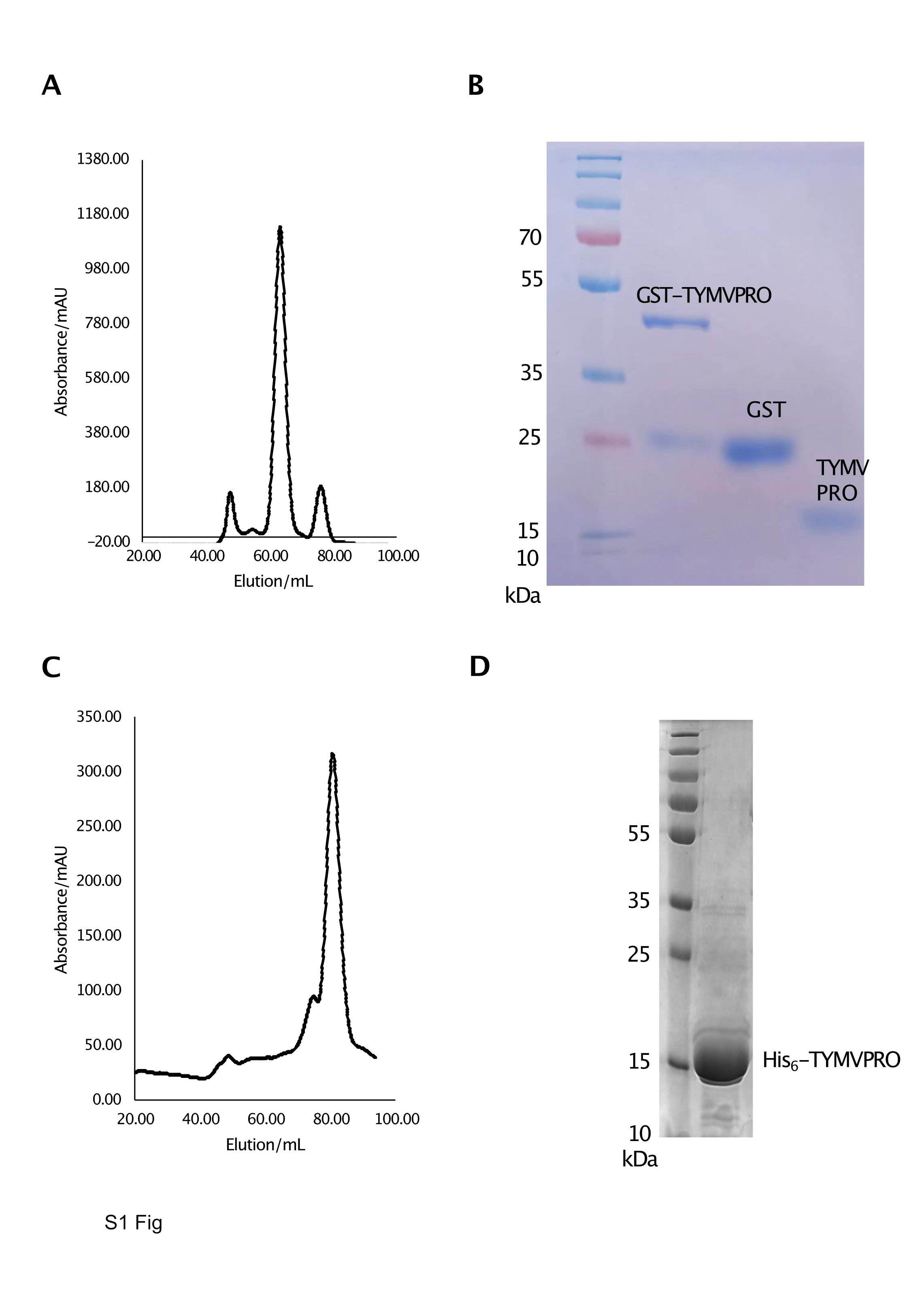

Supplement: S1 Fig — (A) Chromatogram (FPLC) of His6-TYMV PRO using Superdex 75 column (GE Healthcare) equilibrated with 20 mM TRIS, 150 mM NaCl, 2 mM DTT, pH 8.0. The third peak (~78 ml) is TYMV PRO. (B) 12% SDS PAGE gel of purified TYMV PRO without GST tag (cleaved by HRV3c Precision Protease) and the fractions collected from each peak as follows, 1: GST tagged TYMV PRO (~43kDa), 2:GST (25kDa) and 3: TYMV PRO (~18kDa). (C) Chromatogram of His6-TYMV PRO. (D) 12% SDS PAGE gel of purified His6-TYMV PRO (~18kDa). (TIFF) [file ppat.1012899.s001.tiff]

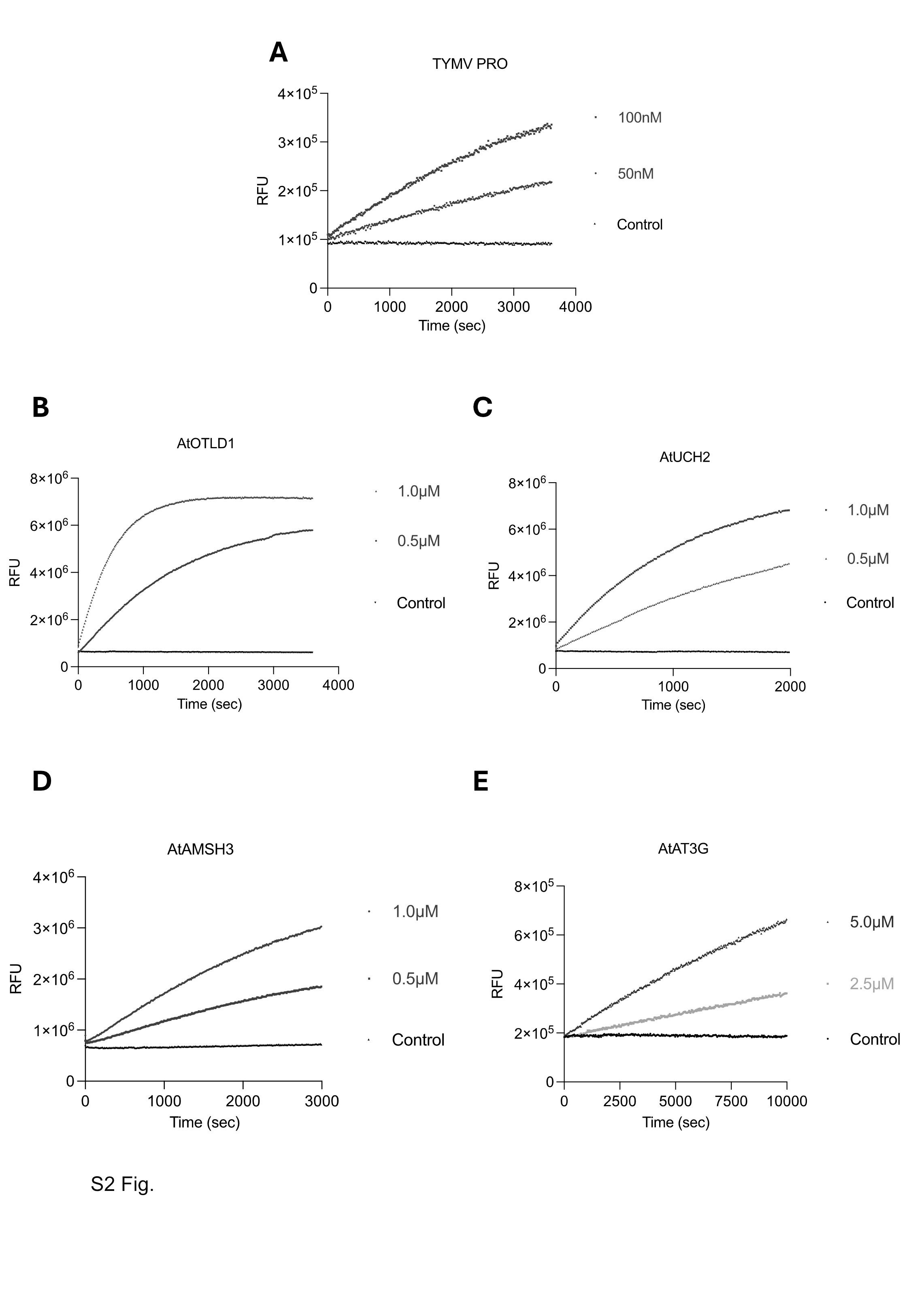

Supplement: S2 Fig — The DUB activity of TYMV PRO (A), AtOTLD1 (B), AtUCH2 (C), AtAMSH3 (D) and AtAT3G (E). The concentrations of enzymes are as follows: dark blue, 1μM; red, 0.5 μM; magenta, 5.0μM; orange, 2.5μM. The black line represents the control (Ub-AMC substrate, 1μM). (TIFF) [file ppat.1012899.s002.tiff]

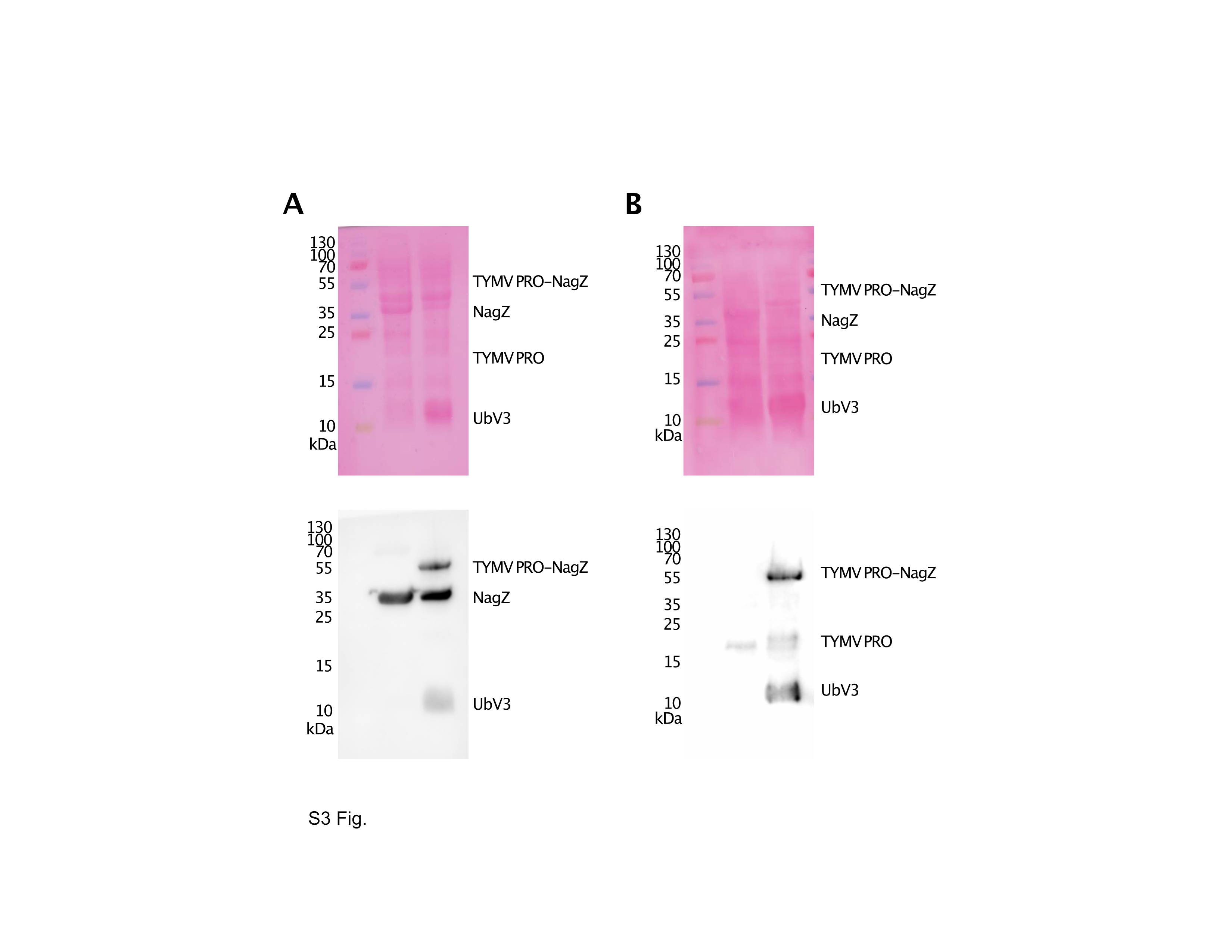

Supplement: S3 Fig — Ponceau S staining of the blots (above) is shown to demonstrate and determine equal protein loading (60 μg). Due to the low amount of protein expressed, the location of TYMV PRO-NagZ on the Ponceau stained gels are estimated based on the positions revealed by the Western blot (below). Cell lysates were analyzed by Western blotting using anti-FLAG tag antibody(A), and anti-Histag antibody (B). (TIFF) [file ppat.1012899.s003.tiff]

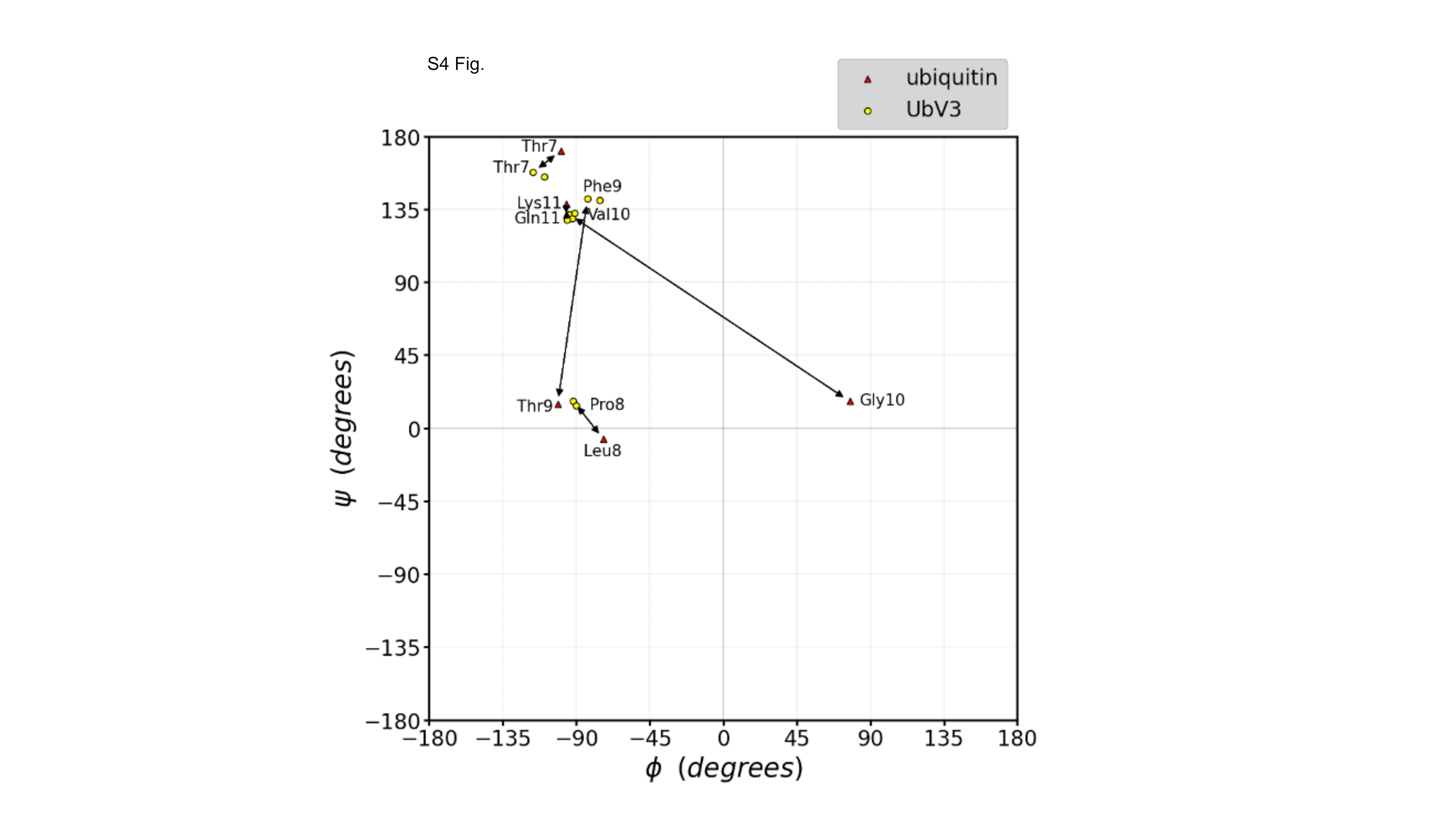

Supplement: S4 Fig — Amino acids of Ub are indicated as red triangle marker (PDB ID: 1UBQ), amino acids of UbV3 were indicated as yellow dot marker. (TIFF) [file ppat.1012899.s004.tiff]

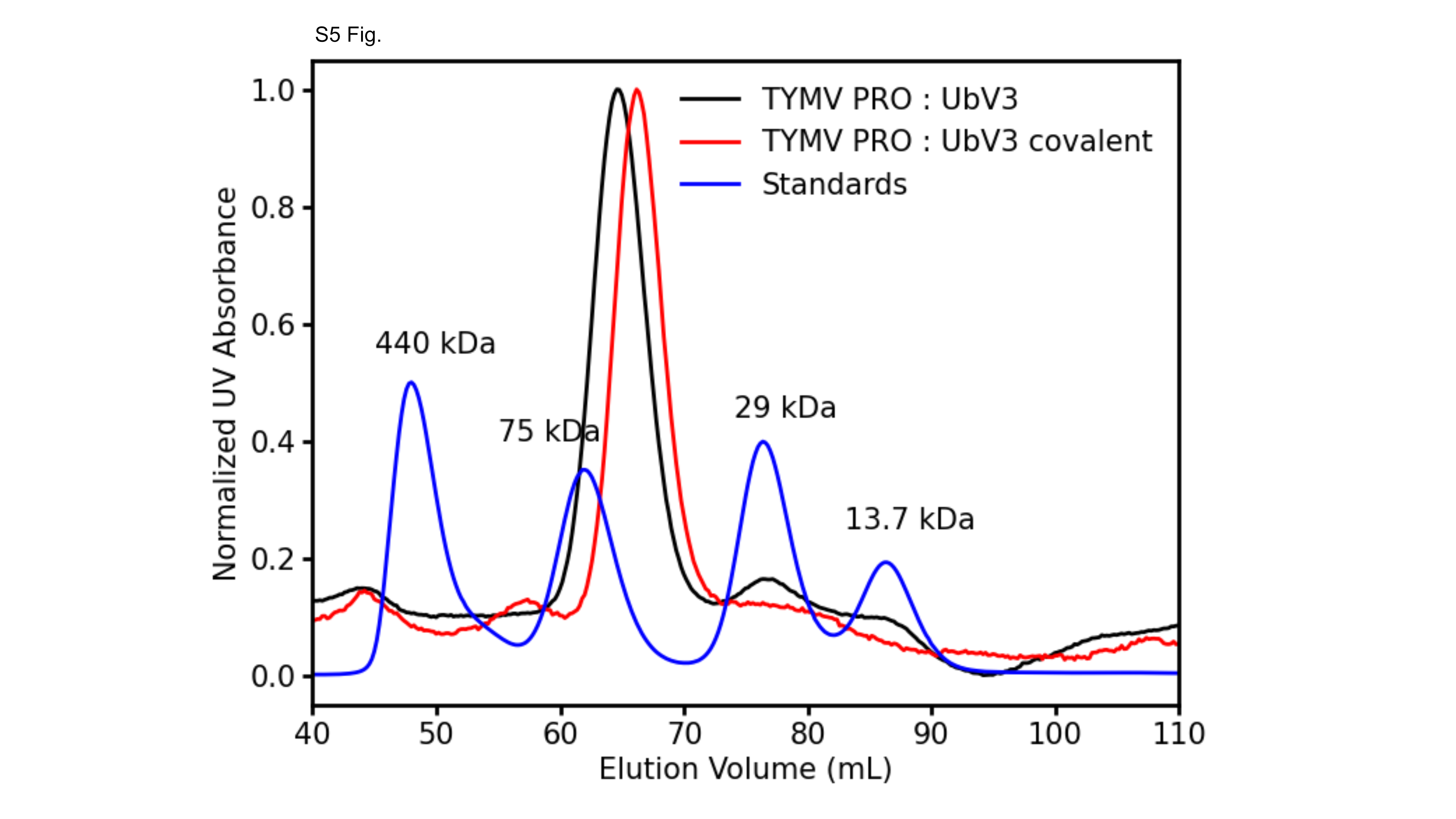

Supplement: S5 Fig — (TIFF) [file ppat.1012899.s005.tiff]

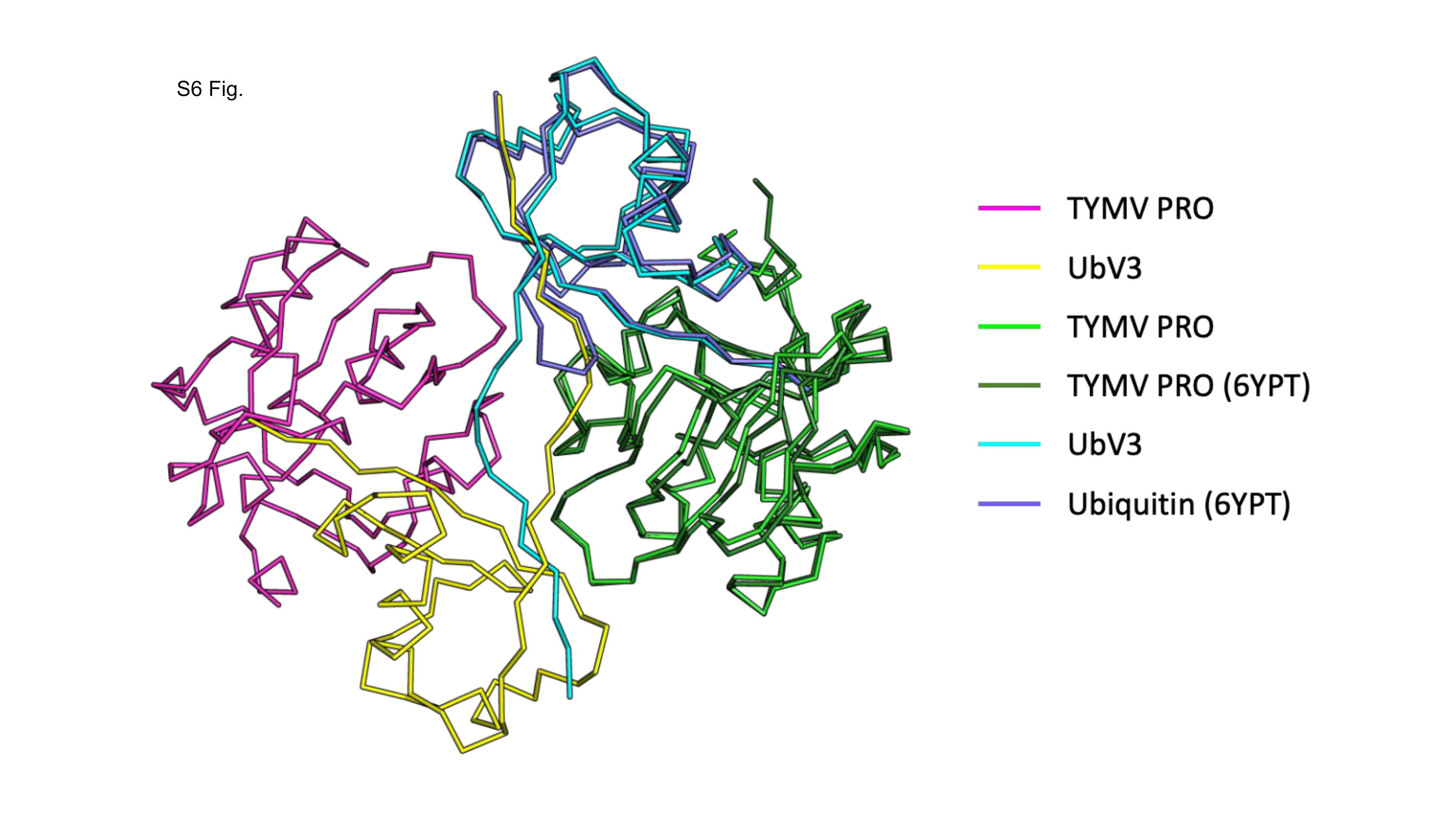

Supplement: S6 Fig — 1:1 complex of 6YPT was superimposed on the half of 2:2 complex of TYMV PRO: UbV3. (TIFF) [file ppat.1012899.s006.tiff]

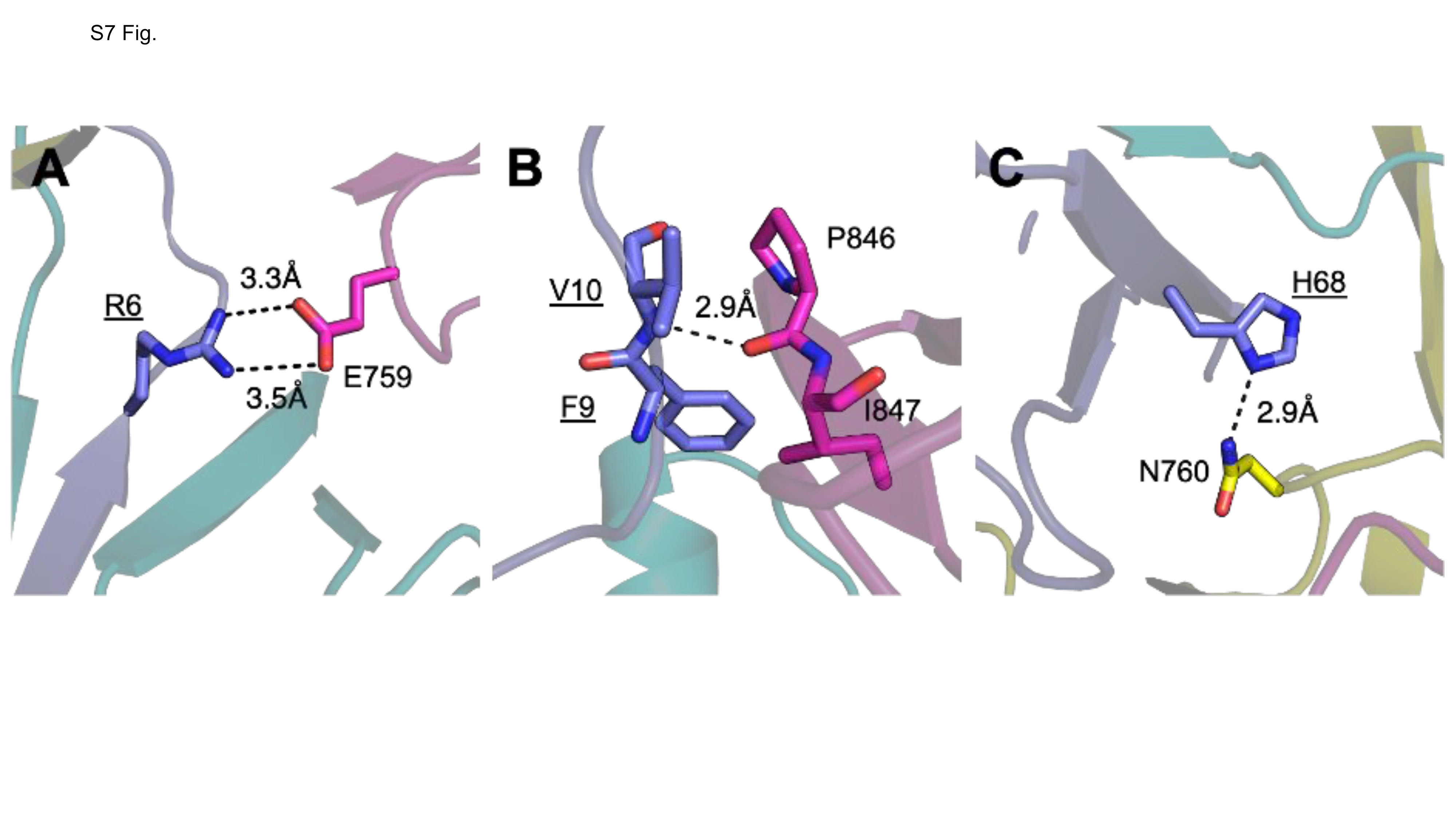

Supplement: S7 Fig — (A) Salt-bridge between UbV3 R6 and TYMV PRO E759. (B) Hydrogen bond between the backbone amide N atom of UbV3 V10 and the carbonyl O atom of TYMV PRO P846. (C) Hydrogen bond between UbV3 H68 and TYMV PRO N760. Possible interactions are indicated by a dashed line with distance. Residues of UbV3 are labeled with underline, and the key residues of TYMV PRO/DUB active site are labelled. (TIFF) [file ppat.1012899.s007.tiff]

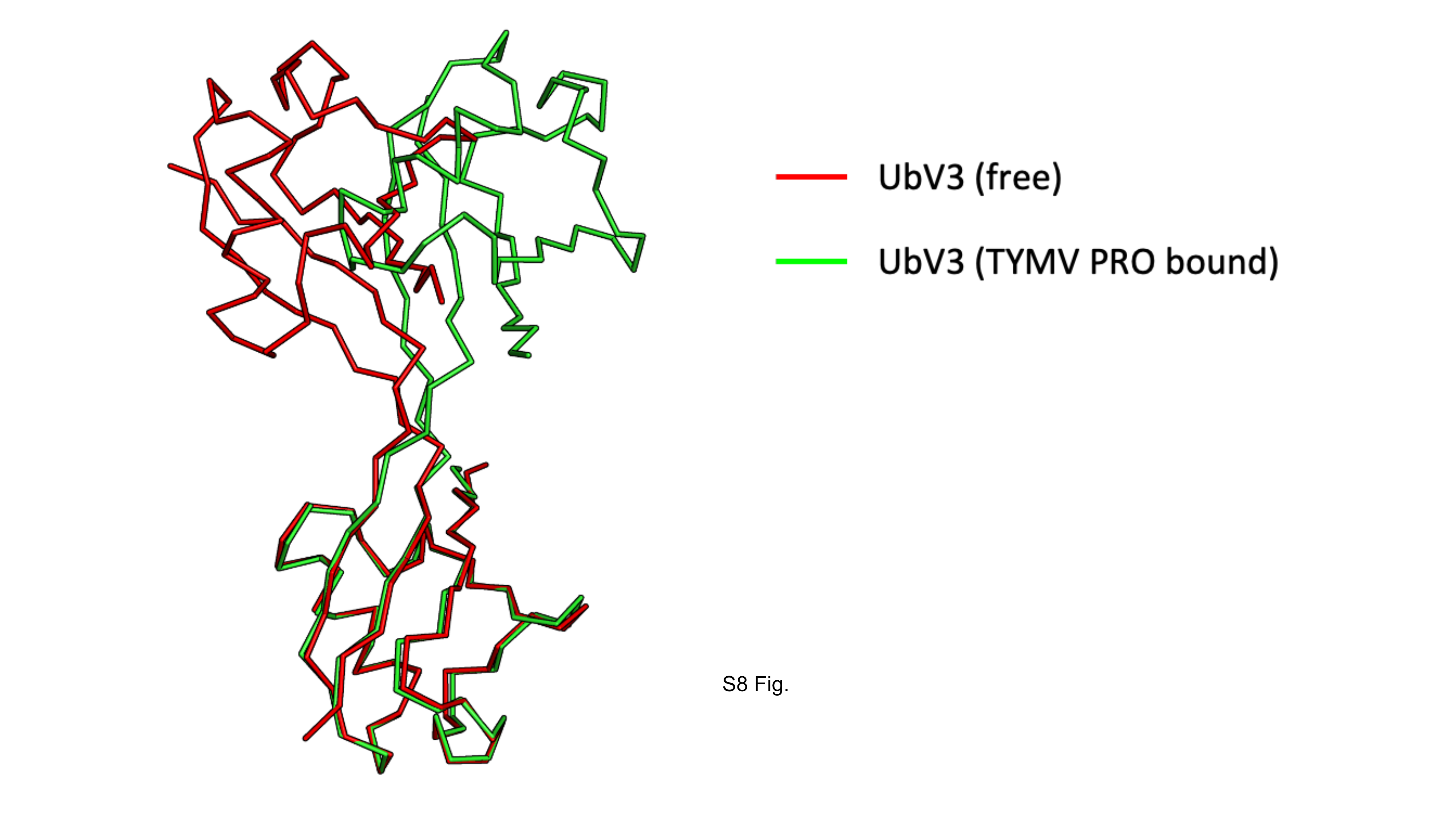

Supplement: S8 Fig — Free UbV3 dimer is colored red, TYMV PRO bound UbV3 dimer is colored green. Single chains of ubiquitin are aligned together to show the orientation of the second ubiquitin chain. (TIFF) [file ppat.1012899.s008.tiff]

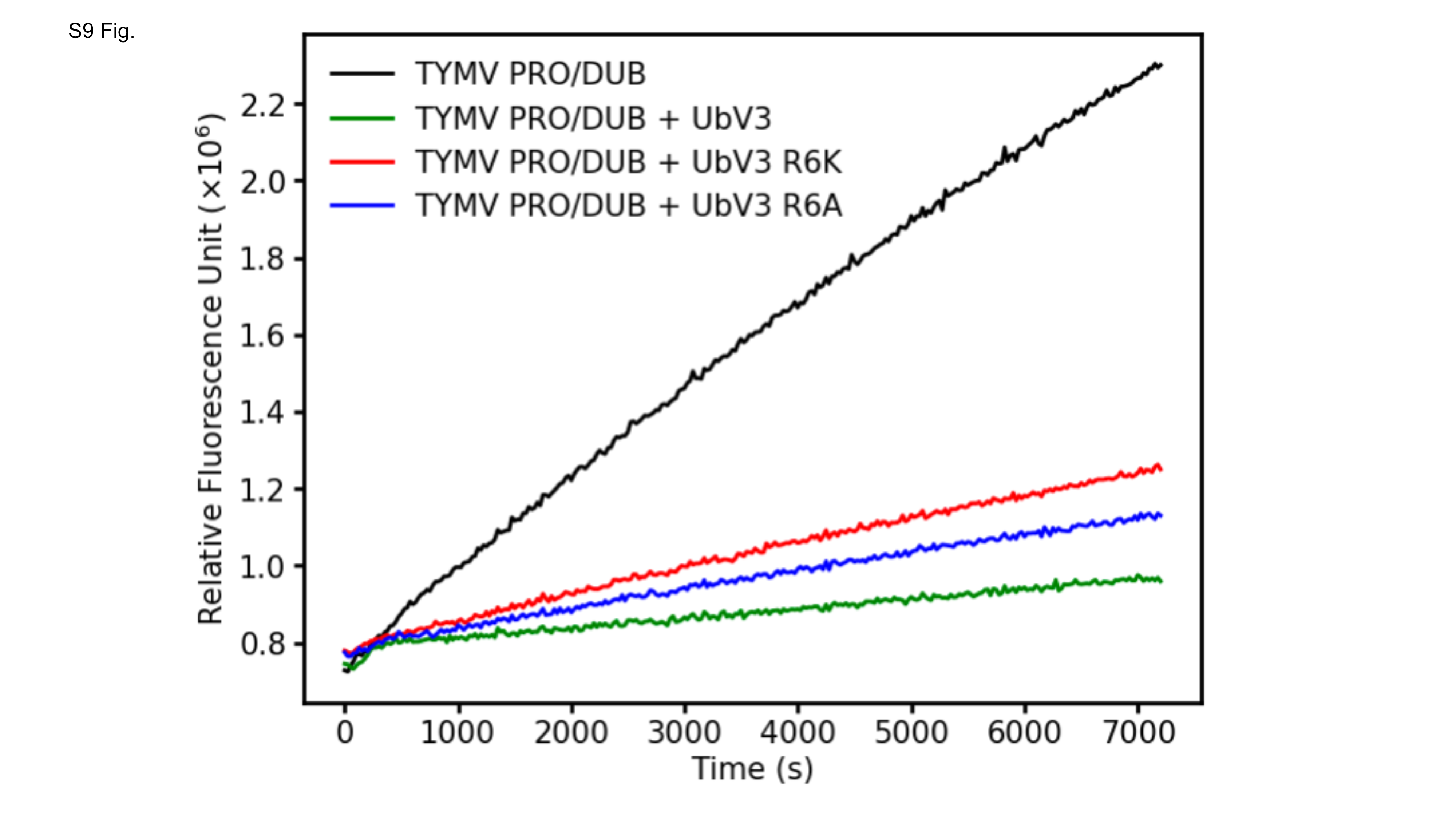

Supplement: S9 Fig — (TIFF) [file ppat.1012899.s009.tiff]

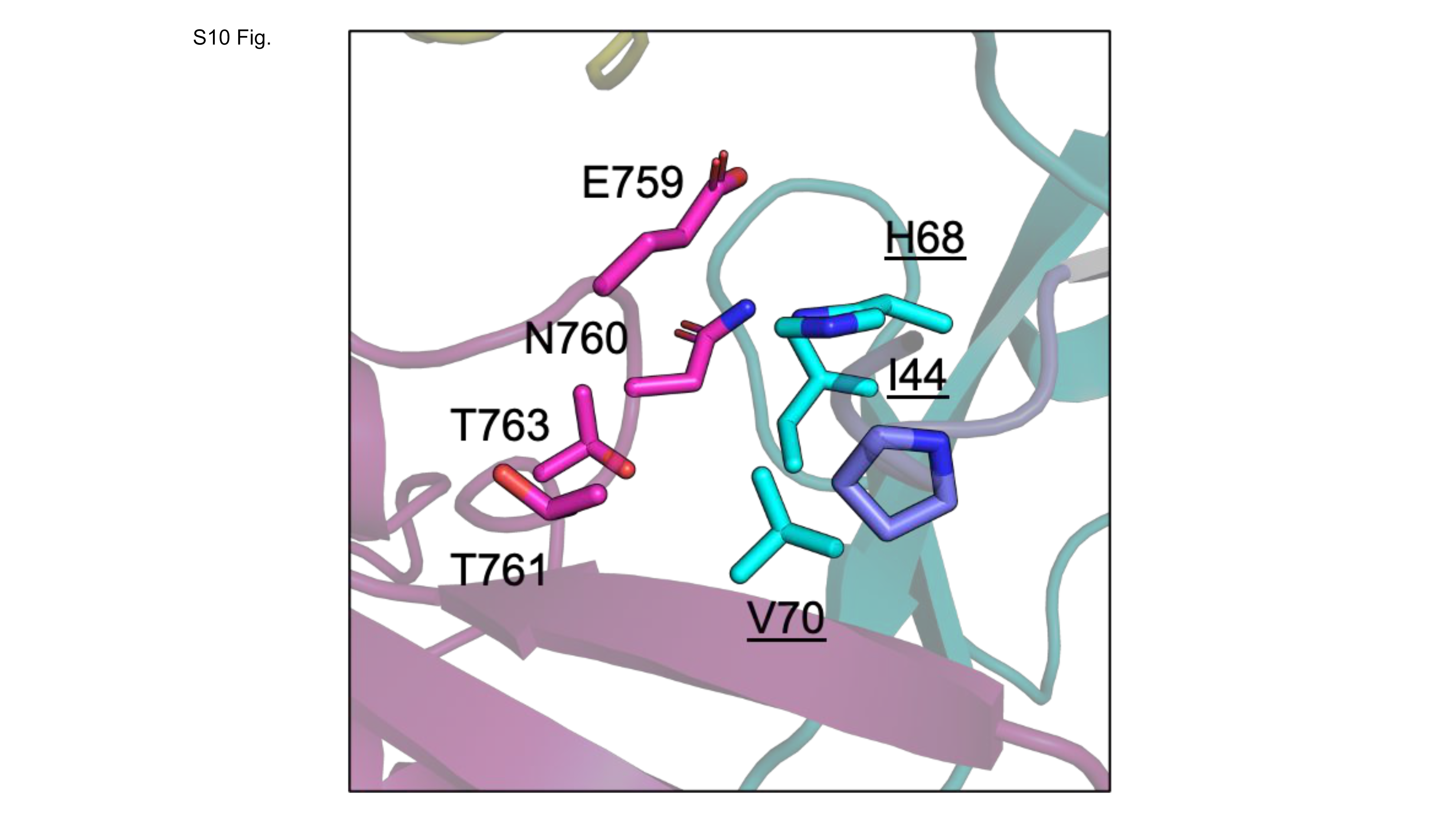

Supplement: S10 Fig — TYMV PRO does not form hydrophobic interaction with the I44 patch of Ub. The two copies of TYMV PRO are coloured magenta and yellow and the monomers of the UbV3 dimer are colored cyan and blue. Some key residues are labelled with residue numbers, underlined for UbV3. (TIFF) [file ppat.1012899.s010.tiff]

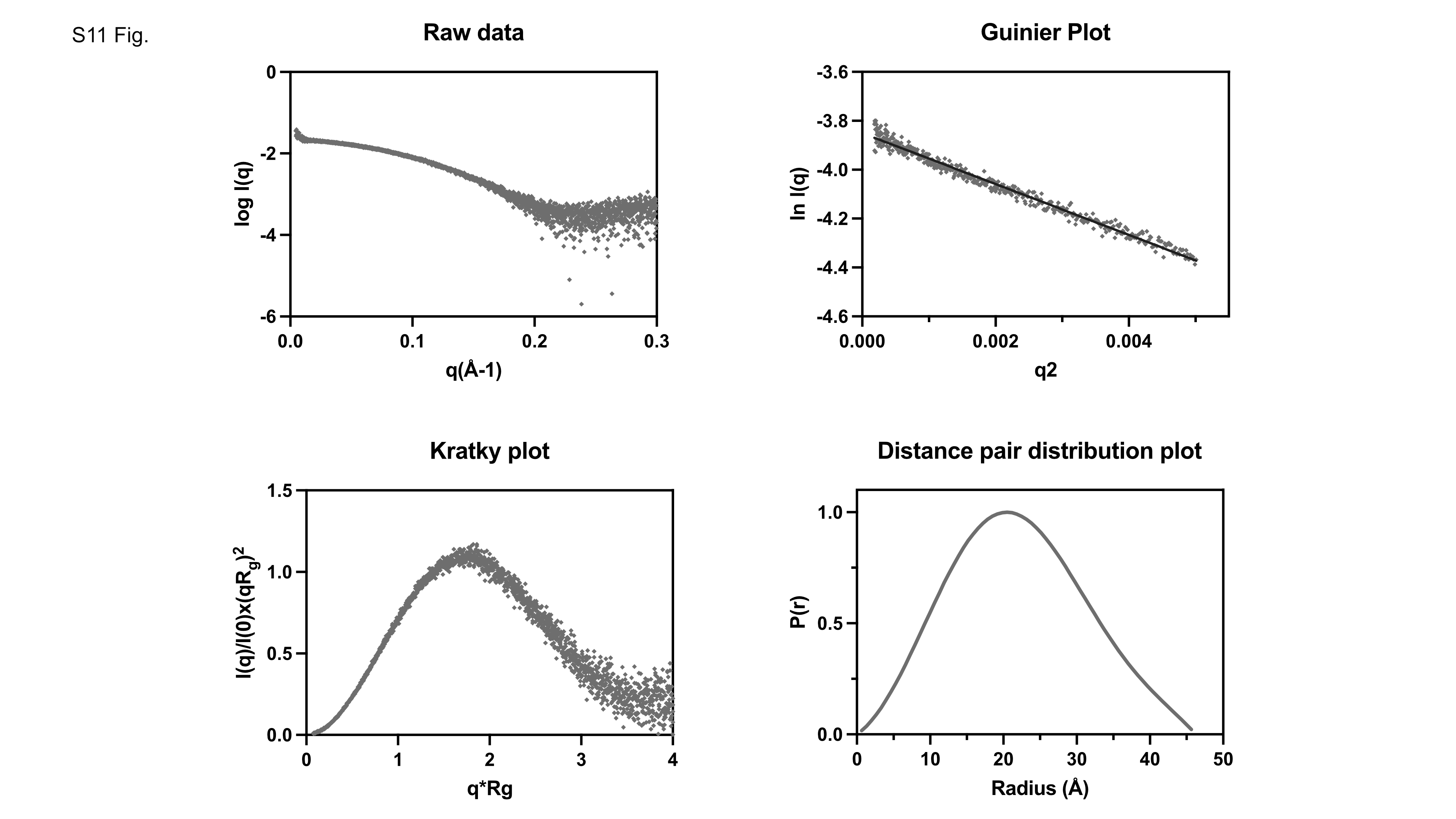

Supplement: S11 Fig — A) Combined scattering data for TYMV PRO showing the relationship between scattering intensity and scattering angle (q = 4πsinϴ/λ). B) Guinier plots illustrate the calculation of Rg and homogeneity obtained from the low-angle area. C) Dimensionless Kratky plots depicting a globular structure as a result of the Gaussian shape of the curve. D) Normalized pair-distance distribution plots for maximal particle dimension (Dmax) determination from the entire SAXS dataset. (TIFF) [file ppat.1012899.s011.tiff]

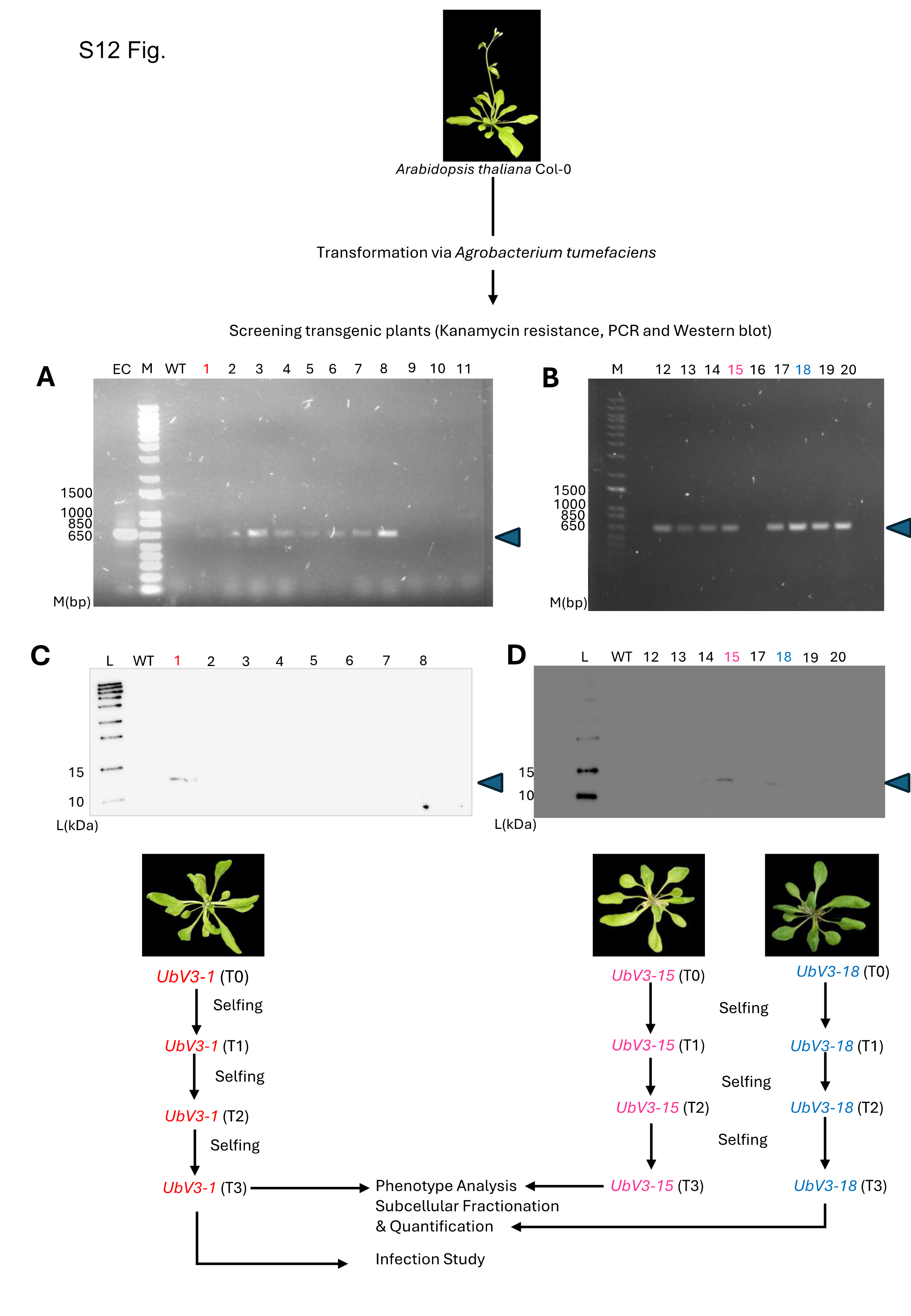

Supplement: S12 Fig — Transgenic plants were generated by Agrobacterium-mediated transformation, and plants that showed kanamycin resistance were further screened via PCR and Western blot analysis for the presence of the UbV expression cassette and protein, respectively. The size of the expression cassette (EC) is 661bp. During the initial screening, 20 plants (A: 1–11 plants and B: 12–20) were screened by PCR, and the resulting amplicons were highlighted by blue arrows (M: DNA molecular weight marker). Selected transgenic lines were screened by Western blot analysis to confirm the presence of FLAG-tagged- UbV3 protein (~12kDa) [L: Molecular weight marker]. Three transgenic lines (UbV3-1, UbV3-15 and UbV3-18) expressed FLAG-tagged UbV3 (C and D). These lines were allowed to self-pollinate. The UbV3-1, UbV3-15 and UbV3-18 plants were selected for phenotypic analysis and subcellular fractionation, and the UbV3-1 line was used for the in vivo infection study (After two successive selfings, the only UbV3-1 transgenic plant lines yielded a stable phenotype (third-generation plants, T3), and all T3 plants expressed UbV3 inhibitor). (TIFF) [file ppat.1012899.s012.tiff]

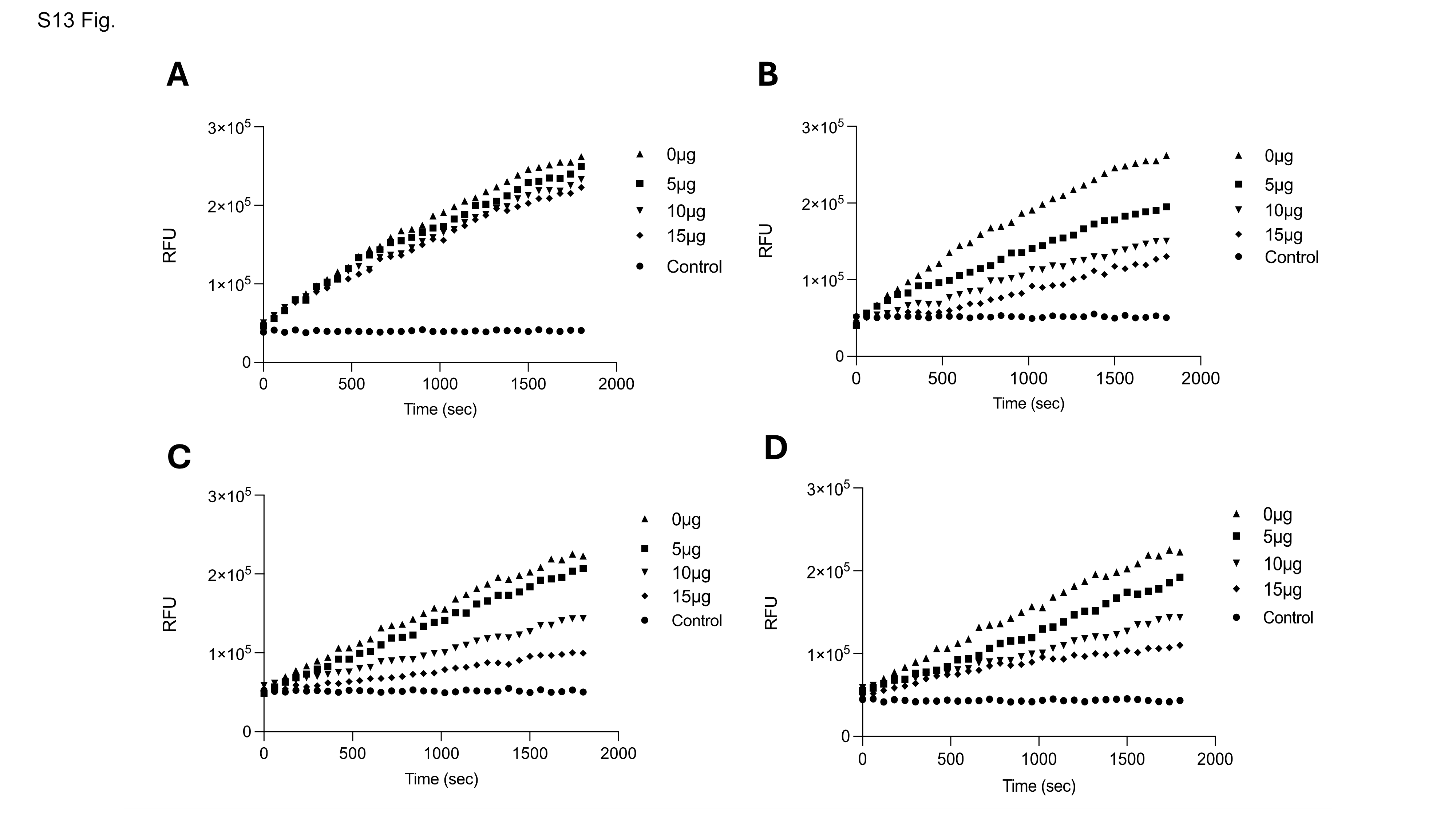

Supplement: S13 Fig — (A) WT A. thaliana protoplast cytosolic fraction and (B-D) transgenic A. thaliana protoplast cytosolic fraction from (B) UbV3-1 (B), (C) UbV3-15, and (D) UbV3-18. The line symbols represent the concentration of the cytoplasmic fraction (μg), and the black circle line represents the 1 μM of Ub-AMC control. (TIFF) [file ppat.1012899.s013.tiff]

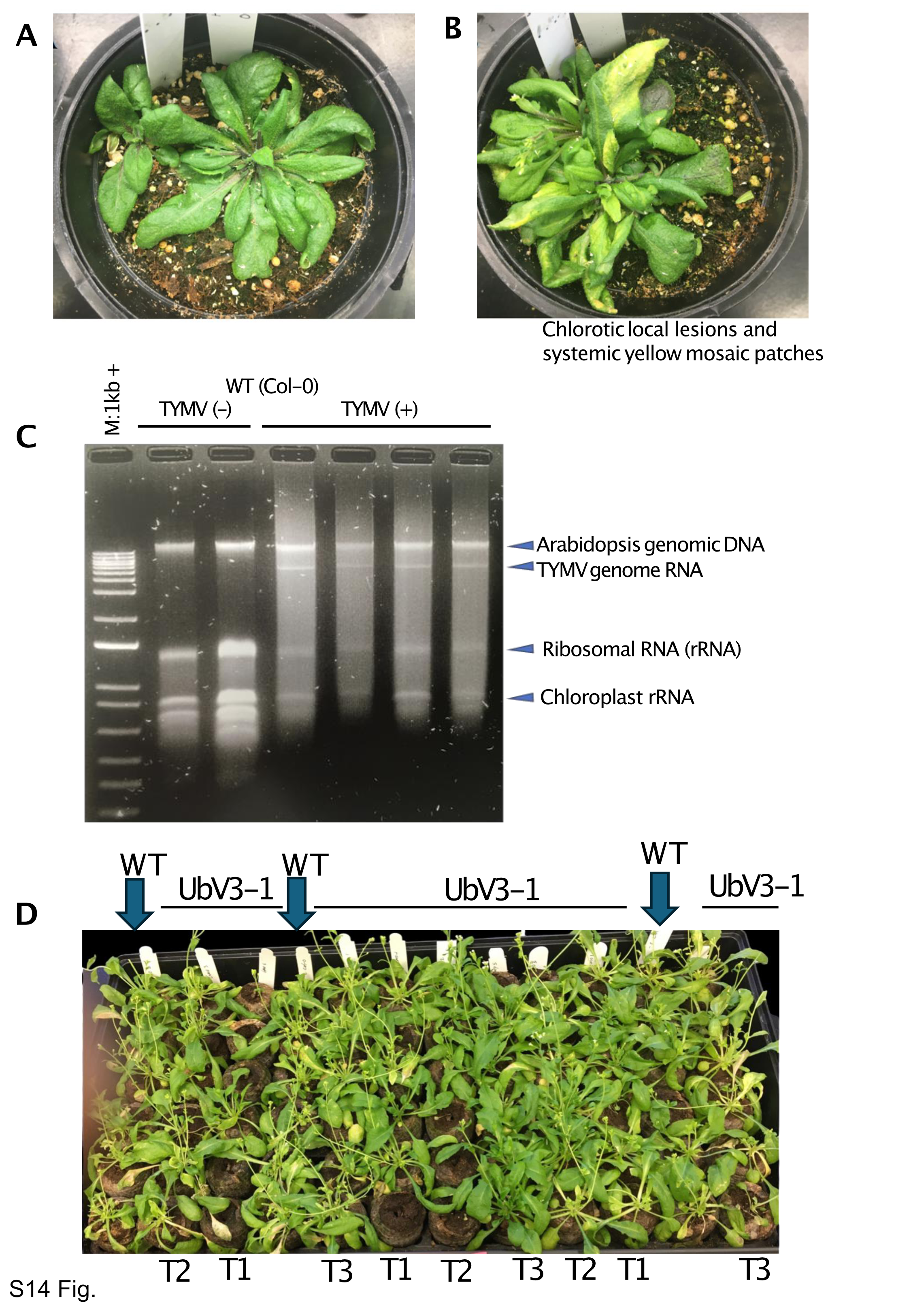

Supplement: S14 Fig — Wild-type A. thaliana Col-0 (A: H2O treatment and B: TYMV infection). (C) Gel electrophoresis of total nucleic acid extracts from wild-type A. thaliana with and without TYMV infection. The rRNA and chloroplast rRNA are reduced after TYMV infection. (D) TYMV infection experiment setup and symptom variability. The WT (A. thaliana Col-0) and Transgenic A. thaliana (UbV3-1) with different generations (T1, T2 and T3) were sprayed with TYMV before flowering and at a late stage (foliar spray). This figure shows the late-stage infection symptoms in plants. Plants were kept under fluorescent lights at ~24°C and 12 h day length. The third transgenic generation plants (T3) were used for the coat protein determination experiment. (TIFF) [file ppat.1012899.s014.tiff]

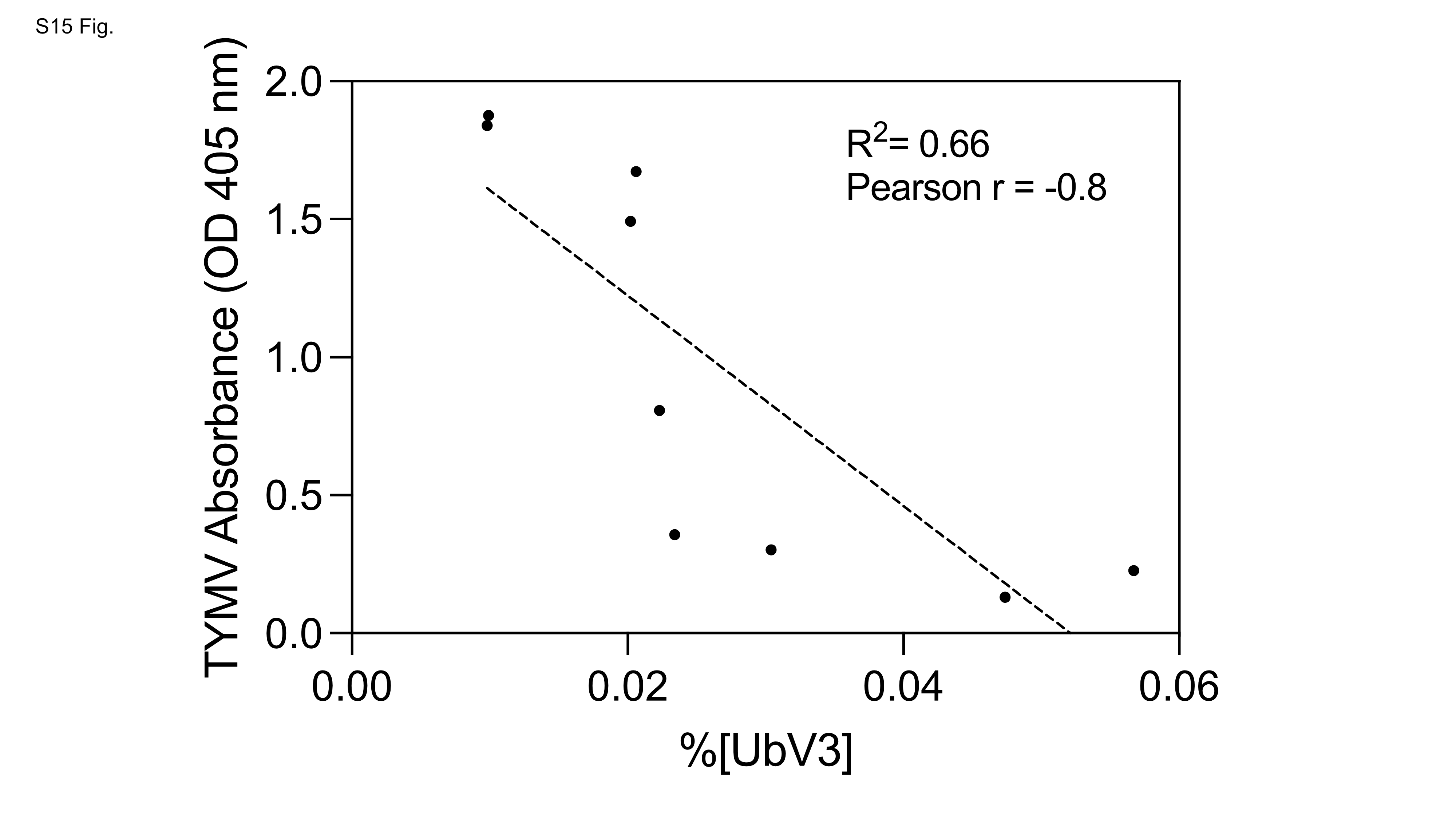

Supplement: S15 Fig — The data were statistically analyzed using Pearson correlation analysis, and a strong negative correlation was observed in the Transgenic line UbV3-1(Pearson r = -0.8). (TIFF) [file ppat.1012899.s015.tiff]

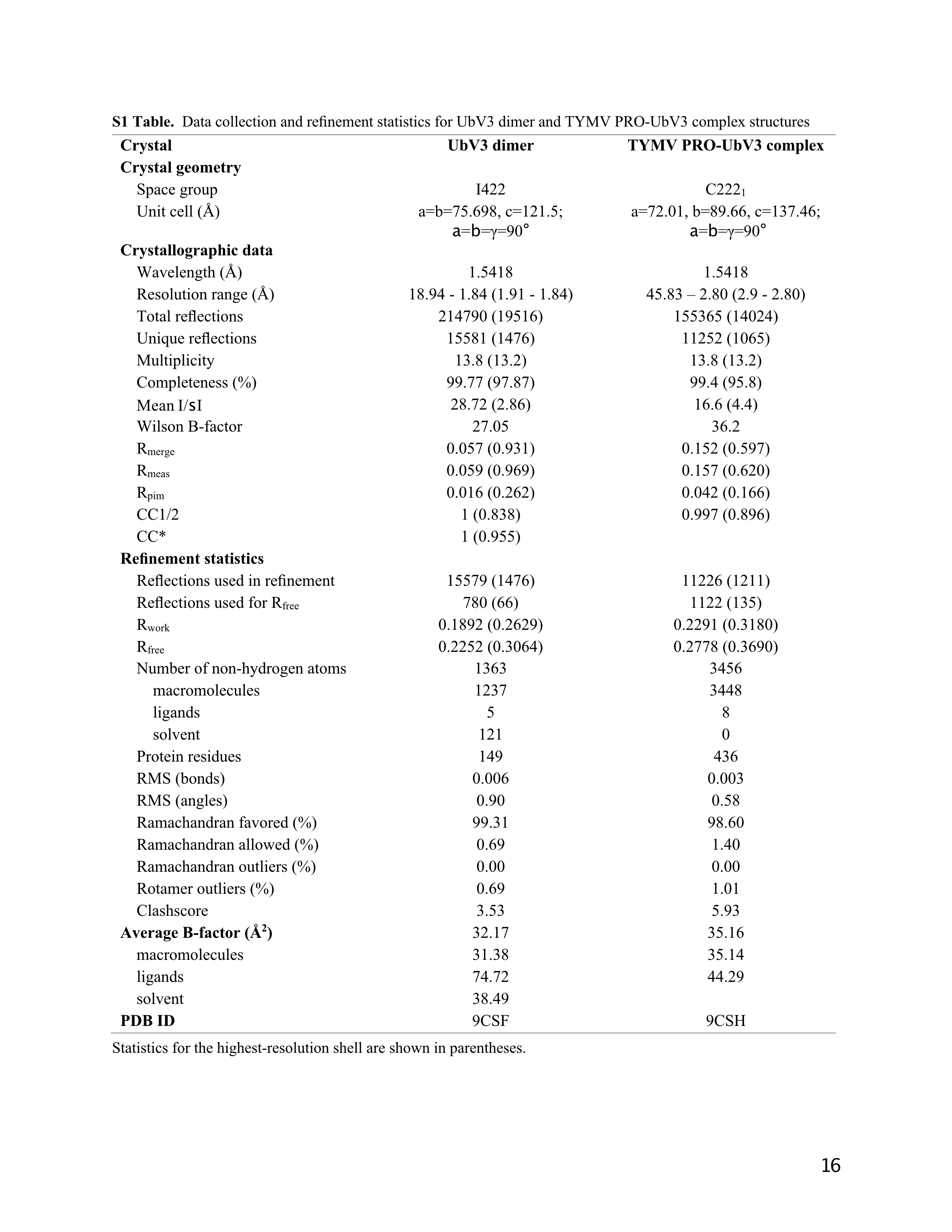

Supplement: S1 Table — (TIFF) [file ppat.1012899.s016.tiff]

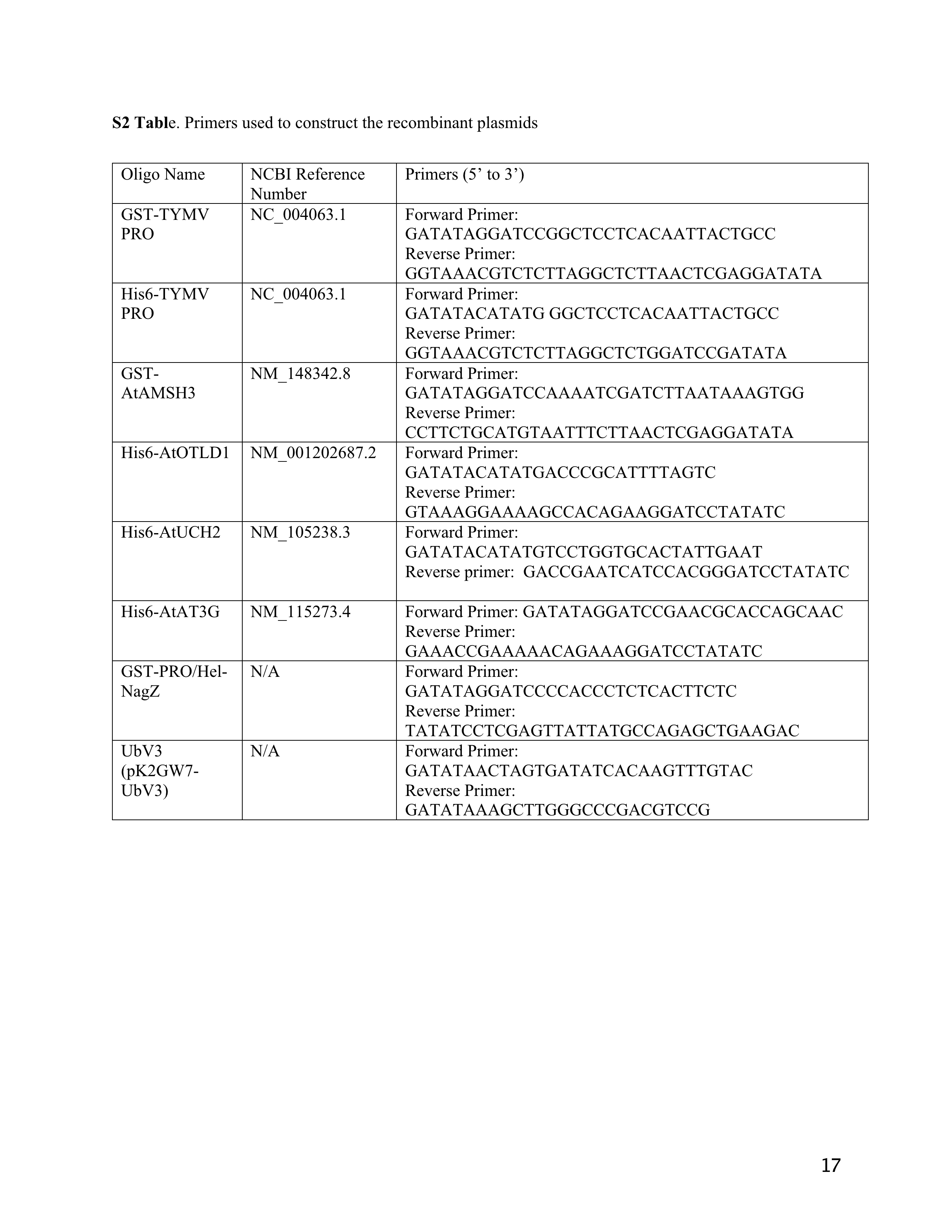

Supplement: S2 Table — (TIFF) [file ppat.1012899.s017.tiff]
